# Transcriptional response to chronic long-access fentanyl self-administration in rat habenula and amygdala

**DOI:** 10.1101/2025.11.25.690517

**Published:** 2025-12-12

**Authors:** Robin Magnard, Daianna Gonzalez-Padilla, Ege A. Yalcinbas, Emma Chaloux-Pinette, Nicholas J. Eagles, Michael S. Totty, Patricia H. Janak, Leonardo Collado-Torres, Kristen R. Maynard

**Affiliations:** 1 Department of Psychological and Brain Sciences, Krieger School of Arts and Sciences, Johns Hopkins University, Baltimore, MD, USA; 2 Lieber Institute for Brain Development, Johns Hopkins Medical Campus, Baltimore, MD, USA; 3 Department of Psychiatry and Behavioral Sciences, Johns Hopkins School of Medicine, Baltimore, MD, USA; 4 The Solomon H. Snyder Department of Neuroscience, Johns Hopkins School of Medicine, Baltimore, MD, USA; 5 Medical Scientist Training Program, Johns Hopkins University School of Medicine, Baltimore, MD, USA; 6 Department of Biostatistics, Johns Hopkins Bloomberg School of Public Health, Baltimore, MD, USA; 7 Kavli Neuroscience Discovery Institute, Johns Hopkins University, Baltimore, MD, USA; 8 Center for Computational Biology, Johns Hopkins University, Baltimore, MD, USA

## Abstract

Fentanyl is a potent synthetic opioid associated with overdose. However, little is known about fentanyl-induced molecular adaptations in the habenula and amygdala, two brain regions implicated in opioid use and withdrawal. We performed bulk RNA-sequencing in the rat habenula and amygdala to identify transcriptomic changes associated with fentanyl intake. Rats self-administered intravenous saline or fentanyl over 22–24 days. Ninety minutes following the final session, brains were collected for transcriptomic profiling. In Hb, we identified 453 differentially expressed genes (DEGs) between saline and fentanyl rats, with upregulated genes associated with synaptic transmission and ionic conductance. In amygdala, we identified 3,041 fentanyl-associated DEGs with upregulated genes implicated in metabolic and vesicular functions. Downregulated genes in both regions were enriched for extracellular matrix functions. Integration of DEGs with single-cell RNA-sequencing data from rodents and humans revealed that fentanyl DEGs were enriched in specific habenula and amygdala cell type markers. Furthermore, fentanyl downregulated DEGs in amygdala were enriched in genes associated with risk for substance use disorders. Together, we define how fentanyl intake alters transcriptional programs in the rat habenula and amygdala, and we link these changes to specific human cell types and risk genes for neuropsychiatric disorders and addiction.

## Introduction

Over the past decade, overdose deaths involving opioids have been rising due to the prevalence of illicit use of synthetic opioids [1–3]. Fentanyl is a synthetic opioid 50–100 times more potent than other μ-opioid receptor (MOR) agonists, such as morphine and oxycodone [4], and it has disproportionately contributed to the rise in opioid-related deaths [2,5,6]. Beyond its potency, additional pharmacological properties that distinguish fentanyl from other MOR agonists include higher lipid solubility allowing more rapid cell membrane penetration and receptor binding [4]. These pharmacological differences have downstream molecular consequences, such as reduced sensitivity to reversal by the MOR antagonist, naloxone, compared to morphine [2,7]. Thus, a deeper understanding of the molecular adaptations induced by fentanyl intake is needed to identify pathways that can be targeted to mitigate its effects.

Opioids cause long-term molecular changes in the brain, especially reward circuits involving the habenula (Hb) and amygdala (Amyg) [8–10]. The Hb consists primarily of glutamatergic neurons, and acts as an “anti-reward” center encoding aversive states by modulating monoaminergic hubs such as the ventral tegmental area (VTA), dorsal raphe nucleus (DRN), and locus coeruleus (LC) [11,12]. The Hb has a high density of MOR [13], especially at the transition between the medial and lateral Hb divisions [14,15]. Optogenetic excitation of MOR-expressing Hb neurons promotes aversive states and elicits avoidance [16]. Hb MOR-expressing neurons have also been shown to mediate the aversive effects of naloxone-precipitated withdrawal [17]. While it is clear that opioid signaling in the Hb mediates distinct functional and behavioral responses, no one has yet investigated how opioids, especially potent synthetic opioids, such as fentanyl, alter the transcriptional landscape of this key hub of reward circuitry.

The Amyg includes several nuclei among which the basolateral amygdala (BLA) and central amygdala (CeA) play an important role in processing emotional valence and affective behaviors [18,19]. The BLA assigns value to environmental stimuli and is critical for associating the negative emotional state experienced during opioid withdrawal with discrete contexts and cues [20–22]. BLA also supports context dependent memories. Song et al., 2019 [23] showed that retrieving morphine-associated withdrawal memories requires BLA plasticity and coordinated activity within a BLA→prelimbic cortex loop. More recently, chronic oral fentanyl consumption and withdrawal were found to impair fear extinction learning and induce persistent hyperexcitability in BLA glutamatergic principal neurons [24]. In contrast, the CeA has been implicated in the negative affective state of opioid withdrawal [18,25]. Lesioning the central nucleus of the amygdala (CeA) blocks morphine withdrawal-induced conditioned place aversion [26]. A population of MOR-expressing neurons in the CeA is activated during fentanyl withdrawal and drives aversive symptoms and negative reinforcement [27]. Transient hyperactivity of this MOR-expressing CeA neuron population is tied to naloxone-precipitated withdrawal in mice subjected to repeated fentanyl injections compared to drug naive mice [27]. Furthermore, opioid-induced hyperalgesia can be produced by repeated fentanyl administration in rodents, and the CeA has been implicated in this aversive experience, which is also associated with chronic fentanyl self-administration [28,29]. While there is mounting evidence for the role of the Amyg in withdrawal and negative states following chronic fentanyl, the molecular adaptations that occur in this region during the trajectory of chronic fentanyl SA remain unexplored.

In rodents, acute or repeated injection of opioids induces gene expression dysregulation in the brain [30–34]. Similarly, chronic self-administration of opioids (i.e. oxycodone, morphine, and heroin) causes widespread molecular changes in reward circuitry nodes, including MOR-rich brain regions like the nucleus accumbens (NAc), dorsal striatum, ventral tegmental area (VTA), prefrontal cortex, and basolateral Amyg, with many of these changes occurring dynamically in different cell types across phases of use (i.e. intake, abstinence, withdrawal, and reinstatement) [9,35–41]. Recent reports have also characterized how chronic long-access fentanyl self-administration alters gene expression in reward circuits [42–44], but these studies did not include the Hb or Amyg. In postmortem human brain, limited studies have identified transcriptomic changes associated with OUD in the dorsal striatum, NAc, and dorsolateral prefrontal cortex [45,46]. However, given that many individuals with OUD engage in polysubstance use [47–49], it is difficult to identify fentanyl-specific transcriptional effects in postmortem human brains, reinforcing the utility of single substance self-administration models in rodents. In induced pluripotent stem cell (iPSC)-derived models, single-cell sequencing of forebrain organoids from patients with OUD showed that repeated exposure to different classes of opioids leads to unique transcriptional responses [50]. Together, these studies highlight the importance of understanding fentanyl-specific transcriptional programs across rodent and human Hb and Amyg.

Here, we performed bulk RNA-sequencing (RNA-seq) in the rat Hb and Amyg following chronic long-access fentanyl self-administration to identify shared and unique molecular alterations in these regions associated with fentanyl intake. In each region, we identified differentially expressed genes (DEGs) between saline and fentanyl rats and evaluated enrichment of these DEGs in cellular processes and molecular pathways. We integrated these data with publicly available single-nucleus (sn) and single-cell (sc) RNA-seq data from rodent and human Hb and Amyg [51–54] to explore whether particular cell types are associated with chronic fentanyl self-administration. Finally, using GWAS summary statistics, we investigated whether Hb and Amyg DEGs for chronic fentanyl self-administration are associated with higher risk for neuropsychiatric disorders, including OUD and other substance use disorders (SUD) [55–60].

## Materials and Methods summary

### Rats

Adult male Sprague-Dawley rats (ENVIGO, Frederick, MD) were used in all experiments (n = 8 fentanyl, n = 11 saline). Rats were single-housed in a temperature and humidity-controlled vivarium on a normal light cycle (12/12 hr. light/dark cycle, lights ON at 7 am) with ad libitum access to food and water. The protocol was approved by the Animal Care and Use Committee of Johns Hopkins University and was conducted in accordance with the National Institutes of Health Guidelines for the Care and Use of Laboratory Animals.

### Drugs

Fentanyl Citrate (Cayman Chemical) was diluted in 0.9% sterile saline at 10 ug/mL. Brevital sodium (Henry Schein) was diluted to 10 mg/mL with 0.9% sterile saline.

### Surgeries

Adult male rats, weighing 250–300 grams at the time of surgery, were anesthetized with isoflurane (3–5% induction, 1–2% maintenance) and administered the analgesic rimadyl (5 mg/kg, SC, Zoetis) and the antibiotic cefazolin (70 mg/kg, SC, West-Ward) prior to catheter implantation. A silastic jugular catheter, constructed as described previously [61], was threaded under the skin from a subcutaneous base in the mid-scapular region and inserted into the right jugular vein [61]. Catheters were flushed daily with ~0.1 mL of sterile saline solution containing 10 mg/mL gentamicin sulfate (VetOne) and 100 IU/mL Heparin (Sagent). Once behavioral training began, catheters were flushed before and after self-administration (and daily on rest days), and were assessed daily for blood return. In the event that blood return was absent, rats were administered 0.1 mL of brevital sodium (10 mg/mL in sterile saline, Henry Schein). If the rat did not become ataxic within 10 seconds, the catheter was considered not patent and the rat was removed from the study.

### Self-administration training

After recovery, rats were trained in standard Med Associates operant chambers housed inside sound-attenuating chambers (Med Associates, St Albans, VT, USA). Operant chambers were fitted with two retractable levers (active and inactive), stimulus lights above each lever, speakers, and a house light. Boxes were controlled using Med-PC IV software (Med Associates, St Albans, VT, USA).

**Fig. 1.**
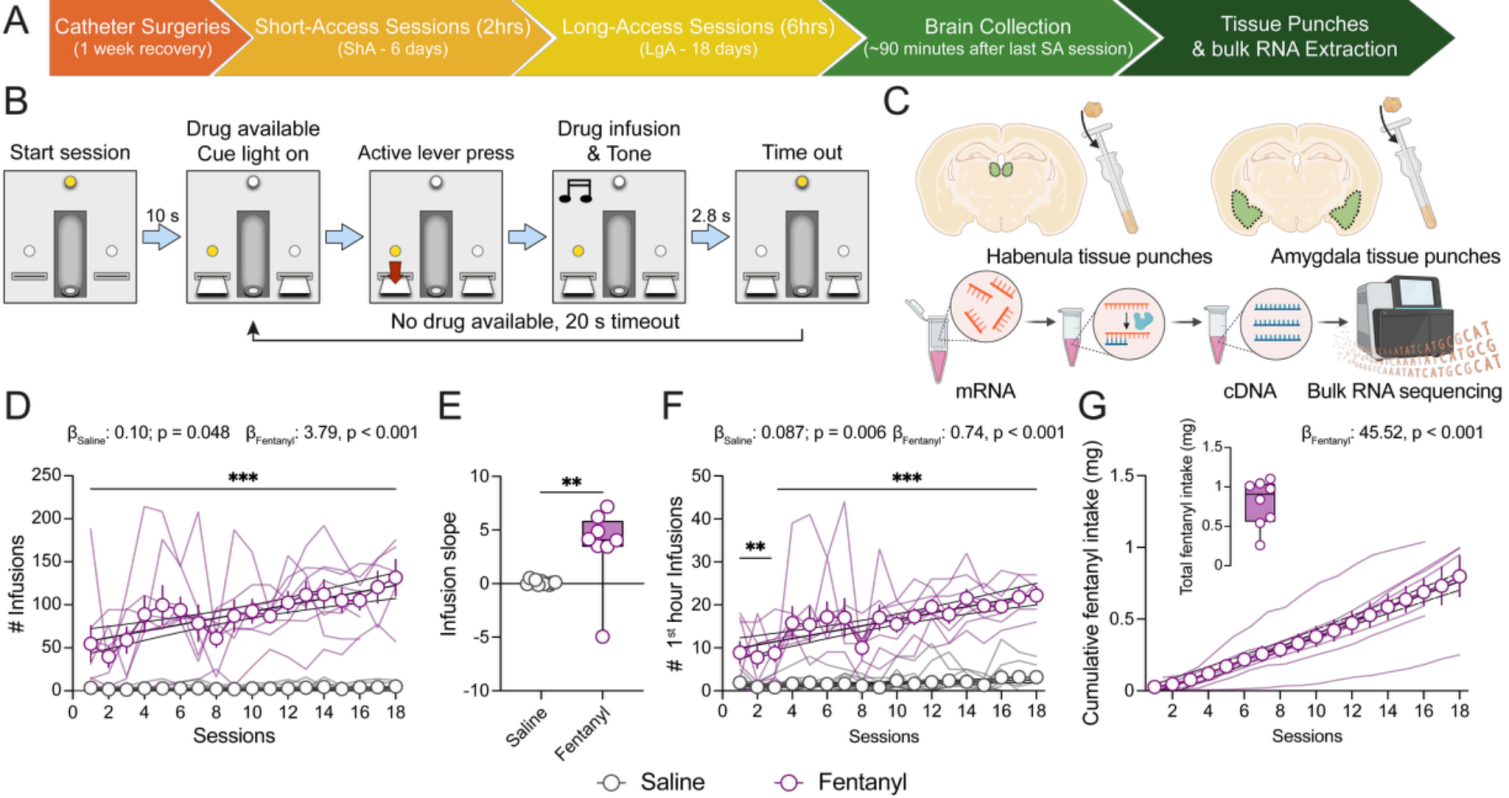
Escalation of fentanyl intake during chronic long-access intravenous self-administration. (**A**) Experimental timeline. Rats were implanted with jugular catheters for intravenous delivery of either saline or fentanyl. Rats first underwent six 2-hour acquisition sessions (Short-Access, ShA), followed by 16–18 daily 6-hour sessions (Long-Access, LgA). (**B**) Intravenous self-administration task design. Fentanyl or saline infusion was contingent upon active lever press on a fixed-ratio 1 (FR1) schedule of reinforcement. Drug or saline availability was signaled by cue light illumination and extinction of the house light. (**C**) Schematic of bilateral habenula and amygdala tissue collection for bulk RNA sequencing. (**D**) Mean number of infusions per LgA session for saline and fentanyl rats (two-way RM ANOVA, substance effect: F_1,17_ = 79.38, p < 0.001; session effect: F_17,273_ = 5.98, p < 0.001; substance x session interaction: F_17,273_ = 5.42, p < 0.001; Student Newman-Keuls post hoc pairwise comparison; ANCOVA substance slope difference: F_1,322_ = 32.07, p < 0.001). (**E**) Individual rat infusion slopes across the LgA sessions (t-test, substance effect t_17_ = 3.14, p = 0.006). (**F**) Mean number of infusions occurring within the first hour of each LgA session for saline and fentanyl rats (two-way RM ANOVA, substance effect: F_1,17_ = 73.59, p < 0.001; session effect: F_17,273_ = 7.93, p < 0.001; substance x session interaction: F_17,273_ = 5.59, p < 0.001; Student Newman-Keuls post hoc pairwise comparison; ANCOVA substance slope difference: F_1,322_ = 32.76, p < 0.001). (**G**) Mean cumulative fentanyl intake (μg) across LgA sessions. Insert: total overall amount of fentanyl intake per rat during ShA and LgA sessions. Data shown as mean across rats ± SEM, superimposed with individual rat data points. Black lines represent linear regression with 95% confidence intervals. Boxes extend from the 25^th^ to 75^th^ percentiles; lines within the boxes represent the median; whiskers indicate the minimum and maximum values. Saline: n = 11 rats; Fentanyl: n = 8 rats. * denotes p < 0.05; ** denotes p < 0.01; and *** denotes p < 0.001.

Rats were trained to lever-press on a fixed-ratio 1 schedule, initially in short-access 2-hour sessions, and then progressing to long-access 6-hour sessions (6 days per week). Both levers were extended for the entire session duration. The stimulus light above the active lever indicated drug (or saline) availability. For fentanyl rats, active lever presses resulted in infusion of 0.5 μg fentanyl dissolved in 50 μl sterile saline delivered over 2.8 seconds [62], while saline rats received an equivalent volume of sterile saline. Infusion was accompanied by a 2.8 second tone, followed by an additional 20 second timeout during which the stimulus light and house light were extinguished; active lever responses during this 22.8 sec interval were not reinforced. After 20 seconds elapsed, the house light and the stimulus light were re-illuminated to indicate availability of drug (or saline). Training occurred for 22–24 sessions, after which the brains were collected. Behavioral data for all the rats included in this study are provided (Figure 1, Figure S1, Table S1, Table S2, Table S3). The escalation ratio, used as an additional metric of infusion escalation across sessions, was calculated by normalizing each rat’s LgA infusion counts relative to its infusion count on the first LgA session.

### Brain tissue extraction and bulk RNA-sequencing

Rats were lightly anesthetized with isoflurane and rapidly decapitated 60–90 minutes after the last long-access self-administration session, and extracted brains were fresh frozen in isopentane and stored at −80 °C. For tissue punching, brains were sectioned into ~2 mm coronal slabs using a rat brain matrix (Stainless Steel Alto Coronal, 1.0 mm matrix, Small Rat 175–300gm), and bilateral tissue punches were collected from the Hb (#39443001RM, Leica Biosystems, nominal diameter 1.25 mm) and Amyg (nominal diameter 1.25 mm) on petri dishes placed in a dry ice bucket. Punched tissue was ejected into tubes sitting in dry ice, and sample tubes were stored at −80 °C until ready for RNA processing. Total RNA was isolated by using TRIzol Reagent homogenization (#15596018, Invitrogen) and chloroform layer separation. RNA was then purified using an RNeasy Micro (#74004, Qiagen) kit with an RNase-Free DNase step (Mat. No. 1023460, Qiagen) according to manufacturer’s instructions. RNA concentration and purity were measured using a NanoDrop Eight (ThermoScientific). RNA quality control assays were performed on the Agilent 2100 Bioanalyzer, and the RNA integrity number for all samples ranged from 7.2 to 8.6 (mean ±SEM = 7.9 ± 0.06).

Ribosomal RNA depletion and library preparation was performed including ERCC spike-ins. Specifically, Illumina Stranded Total RNA Prep with Ribo-Zero Plus Kit was used for library preparation from the habenula samples (10 ng of RNA input), and TruSeq Stranded Total RNA with Ribo-Zero Human/Mouse/Rat Gold Kit was used for library preparation from the amygdala samples (100 ng of RNA input). RNA-sequencing was carried out by Psomagen with the following configuration: ~80M paired-end (PE) reads per sample on an Illumina NovaSeq 6000 S4 (152 bp PE).

### RNA-seq data processing and quality control analysis

Read quality control, read alignment, and gene expression quantification against the rat genome mRatBN7.2 (release 109) was performed using *SPEAQeasy* v87ba0b4 [63]. Lowly expressed genes were filtered and raw counts were transformed to counts per million in a log_2_-scale (log_2_-CPM), normalizing by sample library size and RNA composition [64]. Sample QC metrics (Table S3, Table S4) were examined across brain regions and substances (Figure S2), RNA extraction batches (Figure S3), and number of total self-administration sessions (Figure S4). Quality differed between brain regions, potentially due to the different Illumina kits used for library preparation, and therefore Hb and Amyg samples were analyzed separately in downstream exploratory analyses (Figure S2). Outlier identification of sample QC metrics (Figure S5) and Principal Component Analysis (Figure S6) revealed 4 Hb and 4 Amyg outlier samples (Figure S7), but manual inspection of all QC metrics considered for all these 8 samples supported their inclusion for downstream differential gene expression (DGE) analysis (Figure S7). A final dataset of 16,708 genes across 33 samples (8 fentanyl and 8 saline Hb samples, and 8 fentanyl and 9 saline Amyg samples) was used for DGE analysis.

### Differential Gene Expression (DGE)

**Fig. 2.**
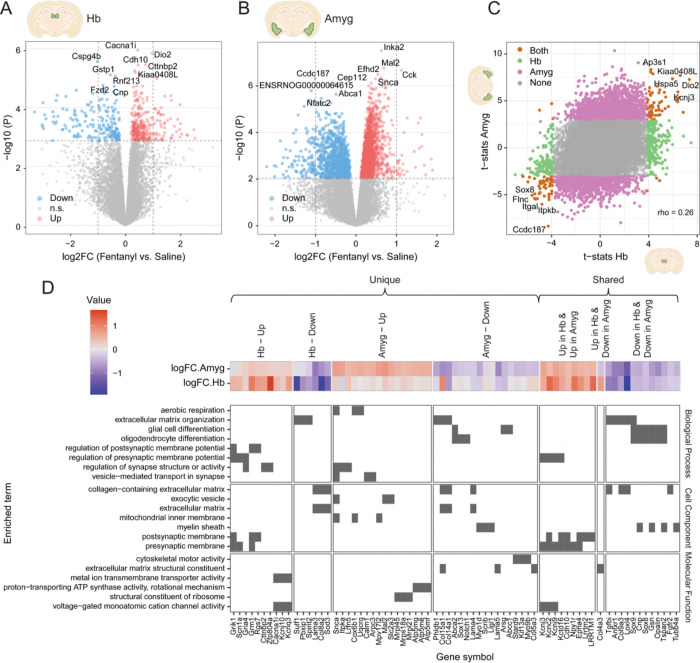
Transcriptional changes and gene ontology (GO) enrichment analysis in Hb and Amyg following chronic LgA fentanyl self-administration. (**A-B**) Volcano plots showing up- and down-regulated genes for fentanyl vs. saline in Hb (**A**) and Amyg (**B**). Horizontal dashed line demarcates significance threshold at FDR=0.05. Vertical dashed lines demarcate log_2_-fold change values of −1 and 1. The top five most significantly up- and down-regulated DEGs are labeled. (**C**) Substance (fentanyl vs. saline) t-statistic correlation plot in Amyg vs. Hb. Pink points are unique DEGs in Amyg. Green points are unique DEGs in Hb. Orange points are shared DEGs in Hb and Amyg. (**D**) Biological processes, cellular components, and molecular functions dysregulated by fentanyl in Hb and Amyg, as identified by Gene Ontology (GO) enrichment analysis of DEGs. Tile plot displays DEG (x-axis) membership to a significantly enriched GO term as a filled tile. Key DEGs per term are shown, categorized by their unique or shared up- and down-regulation in Hb and Amyg. Top heatmap shows DEG mean-centered log_2_FC in Hb and Amyg. Related to Figure S9, Figure S10, Table S5, Table S6, Table S8, Table S9.

DGE between fentanyl vs. saline self-administration and for behavioral traits among fentanyl-administered rats (i.e. the slope of fentanyl infusions across each LgA self-administration session 1^st^ hour, total fentanyl intake across LgA self-administration sessions, and last LgA self-administration session fentanyl intake), was assessed separately in Hb and Amyg using *limma*-*voom* v3.58.1 [65]. In each DGE model, gene expression was adjusted for batch effects and quality control metrics selected based on their correlations with other covariates and their contributions to gene expression variance (Figure 2, Figure S8). Genes with FDR-adjusted *p*-value<0.05 were considered differentially expressed genes (DEGs).

### Functional enrichment analysis

Gene Ontology (GO) biological processes, molecular functions, and cellular components, and KEGG pathways affected by fentanyl self-administration in Hb and Amyg were found by over-representation analysis implemented in *clusterProfiler* v4.10.0 [66] (Figure 2, Figure S10). A total of 14,066 expressed genes, assessed for fentanyl vs. saline DGE, and with available EntrezIDs, were used as the background universe.

### Cell type enrichment analysis

**Fig. 3.**
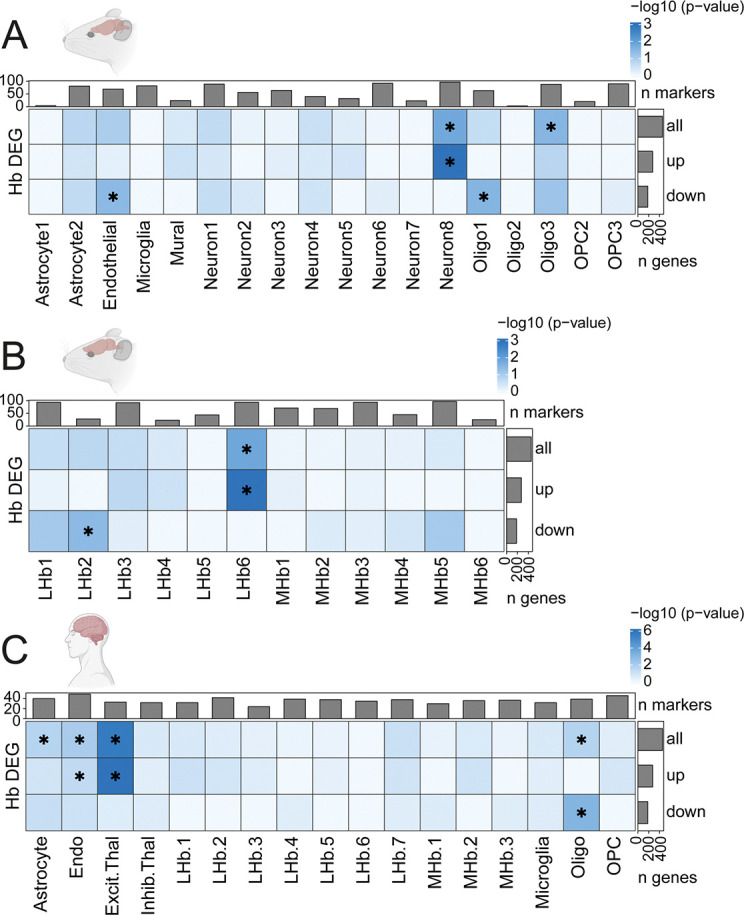
Enrichment of rat Hb fentanyl DEGs in specific human and rodent habenula cell types. (**A-B**) Enrichment of fentanyl vs. saline DEGs (FDR < 0.05) found in rat Hb (y-axis), among the rat orthologues of (**A**) the top 100 markers for broad-level cell type clusters and (**B**) top 100 markers for neuronal subtypes previously identified in mouse habenula by Hashikawa et al. [51] (**C**) Enrichment of rat fentanyl vs. saline DEGs among the rat orthologues of the top 50 markers for broad-level non-neuronal and fine-level neuronal subtypes we previously identified in human Hb/epithalamus in Yalcinbas et al. [52]. Note that mouse (Hashikawa et al. [51]), and human (Yalcinbas et al. [52]) Hb cell type names were not defined to match across datasets. Bar plots along the y-axis depict the number of Hb DEGs that were upregulated, downregulated, or all. Bar plots on top depict the number of rat orthologues used to define each cell cluster. The shade of blue indicates DEG enrichment p-value for a cell type in the −log_10_ scale. * denotes p < 0.05. Related to Figure 2, Figure S11, Table S11, Table S12.

**Fig. 4.**
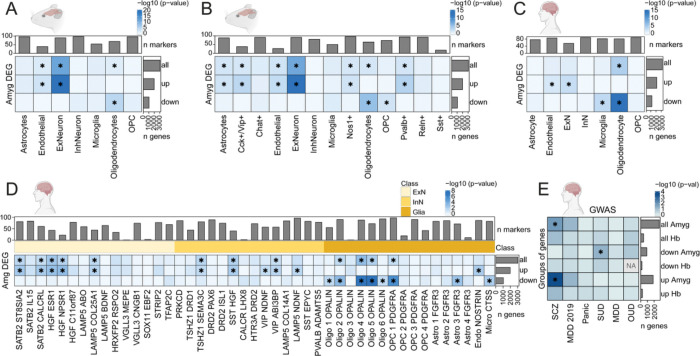
Enrichment of rat Amyg fentanyl DEGs in specific human and rodent amygdala cell types and association of Hb and Amyg rat DEGs with psychiatric disorders. Enrichment of fentanyl vs. saline DEGs (FDR < 0.05) found in rat Amyg (y-axis), among (**A**) the top 100 markers for broad-level and (**B**) fine-level cell type clusters identified in rat Amyg by Zhou et al. 2023 [54] among the rat orthologues of the top 100 markers for (**C**) broad-level and (**D**) fine-level cell types identified in the human Amyg by Yu et al. 2023 [53]. Bar plots along the y-axis depict the number of Amyg DEGs that were upregulated, downregulated, or all. Bar plots on top depict the number of marker genes used to define each cell cluster. The shade of blue indicates DEG enrichment p-value for a cell type in the −log_10_ scale. *p < 0.05. The light yellow, yellow, and orange colors indicate the cell type class: excitatory neuron (ExN), inhibitory neuron (InN), or glia. (**E**) *MAGMA* gene-set enrichment analysis of gene-level Genome-Wide Association Study (GWAS) signals in human orthologs of rat Hb and Amyg fentanyl vs. saline DEGs. The y-axis categorizes fentanyl vs. saline DEGs into upregulated, downregulated, or all, by brain region (Hb or Amyg). The x-axis represents the 5 GWAS datasets that were probed. The shade of blue indicates enrichment p-value in the −log_10_ scale. * denotes FDR-adjusted p < 0.05. Given only 1 human gene whose rat ortholog was downregulated in Hb had OUD-associated SNPs, *MAGMA* couldn’t perform enrichment for this gene set (returned NA). SCZ, schizophrenia; MDD, major depressive disorder; SUD, substance use disorder; OUD, opioid use disorder. Related to Figure 2, Table S13, Table S14.

The cell type specificity of DGE results in rat Hb and Amyg for fentanyl vs. saline self-administration was measured defining marker genes for fine and broad cell types in control rat Amyg [54], control human epithalamus [52] and Amyg [53], and control mouse Hb [51], with the *MeanRatio* method from *DeconvoBuddies* v0.99.0 [67]. Only cell types or clusters with at least 10 cells were considered. Rat orthologs of human and mouse cell type marker genes were obtained with *biomaRt* v2.56.1 [68]. The sets of cell type marker genes in rat were assessed for their over-representation among the rat DEGs in Hb and Amyg applying one-sided Fisher’s exact test (Figure 3, Figure 4). The set of genes assessed for DGE was taken as background (n = 16,708 genes).

### Generalized Gene-Set Analysis of GWAS data

Associations of fentanyl self-administration effects in Hb and Amyg with multiple psychiatric and substance use disorders were interrogated using *MAGMA* v1.10 [69]. This method was provided with Single Nucleotide Polymorphisms (SNP) summary statistics from 5 GWAS datasets to compute gene-level associations with the GWAS disorders under the SNP-wise mean Z-statistics method and using the 1000 Genomes European Phase 3 panel [70] as reference. The GWAS datasets were: Panic Disorder (PD) [59], Opioid Use Disorder (OUD) [56], Substance Use Disorder (SUD) [55], and two for Major Depressive Disorder (MDD) [57,58]. Human orthologs of rat Hb and Amyg DEGs between fentanyl vs. saline were obtained using *biomaRt* v2.61.1 [68], and these were subsequently subjected to a competitive positive one-sided gene-set analysis to find associations at the gene-set level according to the gene-level associations (Figure 4).

### Software

All the analysis code is available at https://github.com/LieberInstitute/fentanyl_rat_hb_amy [71]. Analyses were performed using R versions 4.3.2 to 4.4.0 [72] with Bioconductor versions 3.17 to 3.19 [73]. Visualizations were made using *ggplot2* v3.4.4 and v3.5.1 [74].

## Results

### Chronic long-access (LgA) fentanyl self-administration results in escalation of intake

A week after intravenous catheter implantation, adult male rats were allowed to self-administer saline (n = 11) or fentanyl (n = 8) in 6 short access sessions (ShA, 2 hours) followed by 16–18 long-access sessions (LgA, 6 hours) (Figure 1A). Active lever press resulted in infusion of 0.5 μg of fentanyl dissolved in 50 μl sterile saline, or saline alone (Figure 1B). Sixty to ninety minutes following the final LgA phase, brains were collected for gene expression analysis in Hb and Amyg (Figure 1C). Thus, the molecular changes we present here reflect the combination of same-day and chronic fentanyl.

As expected, during the LgA phase, fentanyl rats showed higher engagement with the active lever compared to the inactive lever (Figure S1A–B), resulting in a greater number of fentanyl infusions and larger infusion slopes compared to saline control rats (Figure 1D-E). This pattern was consistent with results from the escalation ratio, in which we normalized the number of infusions obtained per rat across LgA sessions to the number of infusions from the first session (Figure S1C). Fentanyl escalation across sessions also remained evident when considering only the first hour of each 6-hour LgA session (Figure 1F). On average, rats cumulated a total of 798 μg of fentanyl across LgA sessions (Figure 1G). When combining both short- and long-access phases, total intake ranged from 262 to 1,105 μg of fentanyl per rat (Figure 1G insert). These results demonstrate a progressive escalation of fentanyl intake with repeated exposure.

We next examined the “futile” lever presses, defined as non-reinforced active lever presses made during the tone and timeout periods (Figure 1B). Compared to saline, rats in the fentanyl group consistently made more lever presses during both periods (Figure S1D–E). As these lever presses are not reinforced, they may reflect to some extent an increased motivation to obtain the drug with chronic exposure.

### Chronic fentanyl intake is associated with both shared and unique gene expression changes in Hb and Amyg

The Hb and Amyg are key reward circuitry nodes that have been understudied in the context of molecular adaptations following chronic volitional opioid exposure. To identify transcriptomic changes driven by escalating fentanyl self-administration in these regions, we performed bulk RNA-seq in the Hb and Amyg from individual rat brains collected ~90 minutes after the final LgA session (Figure 1A). Following quality control analysis (Figure S2 to Figure S7), we included 16 Hb (8 saline; 8 fentanyl) and 17 Amyg (9 saline; 8 fentanyl) samples in downstream analyses. We analyzed Hb samples separately from Amyg samples to assess correlations between biological, behavioral, and technical variables, and then performed gene expression variance partitioning to build parsimonious linear models for differential gene expression (DGE) analyses (Figure S8).

Region-specific analyses between saline and fentanyl treated rats revealed 453 differentially expressed genes (DEGs) in the Hb (271 upregulated, 182 downregulated) and 3,041 DEGs in the Amyg (1,988 upregulated, 1,053 downregulated) at FDR < 0.05 (Figure 2A, Figure S9, Table S5, Table S6). In the Hb, top upregulated genes, such as *Cdh10*, *Cttnbp2*, and *Cacna1i*, are mainly involved in synaptic processes and calcium-dependent signaling [75–77] (Figure 2A, Figure S9). In contrast, in the Amyg, top upregulated genes, including *Mal2*, *Cck*, *Snca*, are linked to vesicle trafficking, mitochondrial organization, and ATP synthesis [78–80] (Figure 2B, Figure S9). Among the most significant Hb downregulated genes, *Cspg4b* contributes to oligodendrocyte precursor cell structure and myelination [81], *Gstp1* is central to oxidative stress defense and detoxification [82], and *Rnf213* participates in vascular homeostasis [83] (Figure 2A). In Amyg, the most significant downregulated genes included *Cep112*, involved in maintenance of centrosome integrity [84], *Abca1*, which mediates cholesterol transport and lipid homeostasis [85], and *Nfatc2*, which regulates calcium-dependent transcription and neuroimmune signaling [86] (Figure 2B).

Shared gene expression changes across Hb and Amyg were found (106 shared DEGs), with all but 6 DEGs (*Dact3*, *Plcb4*, *Adarb2*, *Col4a3*, *Abhd17c*, and *Ly6e*) regulated in the same direction (Table S7). Notably, *Col4a3* encoding the ⍺3 chain of type IV collagen, which is involved in extracellular matrix (ECM) organization and vascular integrity [87], was oppositely regulated in Hb and Amyg (Table S7), suggesting possible region-specific vascular remodeling. Among the top shared DEGs (Figure 2C, Table S7) were the stress-related receptors *Crhr2* (downregulated) and *Npy1r* (upregulated), whose opposing changes may suggest acute modulation of stress pathways and compensatory, stress-buffering responses following fentanyl intake. Upregulation of the G protein–activated inwardly rectifying potassium (GIRK) channel subunits *Kcnj3* and *Kcnj9*, suggests an increased inhibitory tone and reduced neuronal excitability within the Hb–Amyg network, immediately after fentanyl self-administration. This pattern aligns with evidence that knockout of these subunits alleviates withdrawal symptoms (see [88] for review), suggesting their upregulation reflects a compensatory adaptation to sustained opioid intake.

Next, we performed functional enrichment analyses using Gene Ontology (GO) terms and Kyoto Encyclopedia of Genes and Genomes (KEGG) pathways to identify biological functions linked to both unique and shared Hb and Amyg DEGs (Figure 2D, Figure S10, Table S8, Table S9). In the Hb, uniquely upregulated genes were primarily associated with synaptic transmission and ionic conductance, including regulators of postsynaptic membrane potential such as *Grik1*, *Scn1a*, and *Gria4*, and voltage-gated cation channels including *Cacna1*, *Kcnj10*, and *Kcnq3* (Figure 2D). In contrast, Amyg-specific upregulated genes were enriched for metabolic and vesicular functions, involving mitochondrial and respiratory components such as *Snca*, *Cox6b1*, and *Uqcrq*, together with vesicle-associated genes like *Calm1* and *Arpc3* (Figure 2D), consistent with increased energy demand and synaptic output. Shared upregulated genes across both regions converged on synaptic organization and excitability, including *Kcnc2*, *Kctd16*, *Cdh10*, and *Epha4* (Figure 2D), reflecting coordinated enhancement of synaptic plasticity within the two structures after fentanyl self-administration. In both regions, fentanyl was also associated with downregulation of extracellular matrix (ECM) genes, such as *Sox9*, *Gsn*, and *Col9a3* (Figure 2D), pointing to reduced extracellular remodeling and glial cell support processes (Table S8, Table S9).

The KEGG pathway enrichment analysis complemented GO results by revealing additional region-specific functional specializations (Figure S10). In the Hb, uniquely upregulated genes were enriched for morphine addiction (*Adora1*, *Gabra4*, *Adcy8*), consistent with enhanced opioid-related signaling within this structure. In contrast, the Amyg showed upregulation of genes linked to oxidative phosphorylation (*Atp6v1e1*, *Atp5mc2*, *Atp6v0e2*), Parkinson disease (*Snca*, *Calm1*, *Slc39a10*), and regulation of actin cytoskeleton (*Arpc3*, *Nckap1*, *Cfl1*), indicating increased mitochondrial metabolism and structural plasticity. However, other Amyg-specific downregulated genes within the same pathway (*Vcl*, *C6*, *Wasf2*) suggest a simultaneous suppression of cytoskeletal reorganization, pointing to a complex balance between synaptic remodeling and stabilization following fentanyl exposure. Finally, pathway analyses of shared DEGs revealed opposite regulation of *Col4a3* within ECM–receptor interaction and *Plcb4* across glutamatergic synapse and gastric acid secretion, highlighting region-specific transcriptional divergence in extracellular and excitatory neurotransmission-related signaling between the Hb and Amyg (Table S8, Table S9).

In addition, we examined transcriptional changes associated with behavior-related metrics among fentanyl rats. We performed DGE for the slope in the number of infusions in the first hour of each LgA session, total overall fentanyl intake across LgA sessions, and fentanyl intake in the last LgA session (Table S1, Table S2, Table S10, Figure S8). No genes were found significantly associated with these behavioral metrics in either the Hb or Amyg. This is likely due to limited statistical power and higher variability when subsetting to the 8 fentanyl rats.

### Enrichment of genes associated with fentanyl intake in human and rodent Hb and Amyg cell types

Bulk RNA-sequencing is a powerful, high throughput approach to identify gene expression changes in individual animals across experimental conditions. However, it is difficult to identify cell type-specific transcriptomic changes from homogenate tissue profiles, which may be important for understanding the molecular impacts of fentanyl in Hb and Amyg. Therefore, to infer cell types most impacted by fentanyl self-administration in these regions, we performed enrichment analyses leveraging 4 publicly available single-cell and single-nucleus RNA-seq datasets across control rodent and human Amyg and Hb [51–54]. Briefly, the top 100 marker genes for broad and fine-level cell types in each dataset were identified as those with the greatest mean expression in the target cell type compared to any other cell type (Table S11, Table S12, Table S13, Table S14). Subsequently, we obtained the rat orthologs of human and mouse cell type markers and assessed their over-representation among fentanyl vs. saline DEGs in rat Hb and Amyg.

First, we assessed enrichment of rat Hb fentanyl-associated DEGs in mouse Hb cell types defined at the broad level by Hashikawa et al. (2020) [51]. We found that upregulated fentanyl DEGs in rat Hb were enriched in a single mouse Hb neuron population (Neuron 8), while downregulated rat fentanyl DEGs were enriched in endothelial cells (Figure 3A, Table S11). To better understand the identity of Neuron 8, we performed a second enrichment analysis using fine cell type annotations for mouse LHb and MHb subpopulations (Figure 3B, Table S11) [51]. Upregulated rat Hb fentanyl DEGs were enriched in a specific mouse LHb subpopulation (LHb.6) marked by expression of *Kcnmb4*, *Fam101b*, *Chrm2*, *Sv2c*, and *Gpr151*. We also observed enrichment of downregulated fentanyl DEGs in mouse LHb.2, marked by expression of *Arpp21*, *Cacna2d1* and *Slc6a1*, at this fine cell type resolution. Next, to evaluate whether rat fentanyl DEGs might be enriched in orthologous Hb subpopulations identified in the human brain, we ran an enrichment test across LHb and MHb neuronal populations and glial cell types from our previously published human Hb snRNA-seq dataset [52] (Figure 3C, Table S12). We found that upregulated and downregulated rat fentanyl DEGs registered to human glial cell types, including astrocytes, oligodendrocytes, and endothelial cells. We did not see fentanyl DEG enrichment in any human Hb neuronal subpopulations, likely due to limitations in power as a result of the small number of LHb and MHb neurons captured in this human dataset. Interestingly, human Hb LHb.2 and LHb.7 subpopulations show high expression of *KCNMB4, GPR151, and CHRM2* (Figure S11A–C), suggesting these cell types may be conserved with mouse LHb.6, which showed an enrichment of upregulated fentanyl DEGs (Figure 3B, Table S11). Human LHb.2 and LHb.7 also highly express *OPRM1* compared to other human LHb subpopulations (Figure S11D), suggesting these may be fentanyl-sensitive LHb populations conserved across species.

Next, we performed similar enrichment analyses of rat Amyg fentanyl-associated DEGs in previously identified Amyg cell types from rat [54] and human [53] snRNA-seq datasets. At the broad cell type level when comparing within species, we found that upregulated rat fentanyl DEGs were enriched in rat endothelial cells and excitatory neurons, while downregulated fentanyl DEGs were enriched in oligodendrocytes (Figure 4A, Table S13). At the fine cell type level, upregulated fentanyl DEGs were still most strongly enriched in excitatory neurons, while downregulated DEGs remained enriched in oligodendrocytes and oligodendrocyte precursor cells (OPCs). However, at the finer level, we also observed enrichment of upregulated fentanyl DEGs in astrocytes and several inhibitory neuron populations, including *Cck*+/*Vip*+, *Nos1*+, and *Pvalb*+ (Figure 4B, Table S13). Notably, *Cck* is one of the top 5 upregulated genes following chronic fentanyl intake in Amyg (Figure 2). Enrichment analyses in human Amyg cell types largely agreed with findings in rodents. At the fine cell type level, upregulated rat fentanyl DEGs were enriched in Endo_NOSTRIN populations and several different excitatory subpopulations in the basolateral amygdala (BLA) expressing *HGF*, *SATB2*, and *LAMP5* (Figure 4C, Table S14). Similar to the rodent analysis, upregulated rat fentanyl DEGs were also enriched in several human inhibitory cell types, such as *SST*+ and *VIP*+ subpopulations, and *TSHZ1*+ intercalated neurons. Of particular interest, *TSHZ1*+ intercalated neurons receive input from VTA dopamine neurons and highly express dopamine receptor 1 (*DRD1*) and *OPRM1* [89,90]. Downregulated rat fentanyl DEGs were enriched in several human glial cell types, including specific populations of oligodendrocytes, OPCs, astrocytes, and microglia (Figure 4D, Table S14).

Finally, we asked whether fentanyl DEGs in Hb and Amyg overlapped with genes associated with risk for various neuropsychiatric disorders, including substance use disorder (SUD) and opioid use disorder (OUD), as identified by genome wide association studies (GWAS) [55–60]. For Amyg, we found that upregulated fentanyl DEGs were enriched in schizophrenia (SCZ)-associated risk genes, while downregulated fentanyl DEGs were enriched in SUD-associated risk genes. No enrichment was observed for fentanyl DEGs in Hb (Figure 4E). Taken together, we identify the enrichment of fentanyl-associated genes in rodent and human Hb and Amyg cell types and demonstrate overlap of these genes with risk genes for SUD in Amyg.

## Discussion

While several studies have examined transcriptomic changes associated with opioid exposure across brain reward structures (e.g. NAc, VTA, dorsal striatum, prefrontal cortex), fentanyl-induced transcriptional programs in the Hb and Amyg have not yet been investigated. Here, we established a chronic long-access fentanyl self-administration paradigm in which rats show escalating fentanyl intake. We performed bulk RNA-seq on individual rats following the last self-administration session and identified both unique and shared DEGs in the Hb and Amyg, many of which were implicated in distinct functional pathways. We integrated fentanyl-associated DEGs with sc/snRNA-seq data from rodent and human Hb and Amyg and identified neuronal and glial cell types enriched in fentanyl DEGs. Finally, we assessed the overlap of fentanyl DEGs with genes associated with risk for neuropsychiatric disorders, including SUD, and found enrichment of SUD risk genes among downregulated fentanyl DEGs in Amyg.

Sixty to ninety minutes after the last session of chronic long-access fentanyl self-administration, we observed both distinct and convergent transcriptional adaptations in the Hb and Amyg. In the Hb, we found upregulated genes related to synaptic transmission and ionic conductance, suggesting heightened excitability, whereas downregulated Hb DEGs involved myelination, vascular regulation, and oxidative stress defense indicating weakened structural and metabolic support. The Amyg, by contrast, showed enrichment for mitochondrial activity, ATP synthesis, and vesicular pathways, consistent with increased energy demand and neurotransmitter release, alongside reduced expression of genes governing cytoskeletal organization, lipid metabolism, and neuroimmune balance. Despite some regional differences, both regions exhibited coordinated upregulation of genes promoting synaptic organization and excitability, accompanied by suppression of ECM and glial-related genes. These findings align with postmortem human studies of individuals diagnosed with OUD, which reveal disrupted synaptic plasticity, ECM and neuroimmune-related functions in the PFC and NAc [45]. Additionally, neuroinflammatory and metabolic alterations across neuronal and glial populations were also observed in the dorsal striatum of individuals who died from opioid overdose [46]. Collectively, these findings point to maladaptive synaptic plasticity as well as altered metabolic and glial homeostasis following chronic opioid volitional intake.

The Hb and Amyg fentanyl-induced transcriptional changes we observed in this study closely align with other recent fentanyl transcriptomic studies. Notably, Wood et al. [91] examined DEGs in the rat CeA following fentanyl exposure. Among the 2,735 genes common to their dataset and our Amyg dataset, only seven (*Tuba4a*, *Atrnl1*, *Ift140*, *Arl8b*, *Ndufa10L1*, *Ndrg4*, *Bhlhe22*) were significantly altered in both. These genes are implicated in functions such as cytoskeletal organization, mitochondrial metabolism, and signal transduction. Limited DEG overlap may stem from differences in sample size, but also likely differences in experimental design: Wood et al. investigated perinatal fentanyl exposure followed by adult self-administration (3 h/day for 12 days) with tissue collected 24 h after the final session, whereas our study examined adult-onset, long-access self-administration (2 h/day for 6 days + 6 h/day for 16–18 days) with tissue collected 90 min post-session. Uchegbu and coworkers [92], found that prenatal fentanyl exposure in mice led to upregulation of myelin- and glia-related genes in the Amyg whereas these pathways were downregulated in our dataset, suggesting opposing transcriptional adaptations between developmental and adult exposure. Complementing these transcriptional studies, an *ex vivo* calcium imaging experiment in rat showed that long-access fentanyl self-administration promotes a reduction in central Amyg activity after a 30 days abstinence period and a shift from acute tolerance to chronic hypersensitivity to fentanyl exposure [93]. These neuronal adaptations likely reflect underlying transcriptional remodeling that disrupts Amyg excitability over time and may drive fentanyl withdrawal symptoms and relapse.

Our dataset also partially overlaps with transcriptomic changes in the VTA after chronic fentanyl self-administration [43]. Our Amyg dataset showed a large and concordant overlap with the mouse VTA (285 shared DEGs), with most shared genes downregulated in both regions and involved in metabolic, mitochondrial, and myelin-related pathways, together with a coordinated upregulation of neuronal excitability and synaptic plasticity genes. While Amyg showed broad overlap with the VTA, the Hb exhibited a distinct transcriptional profile. Among the 58 genes significantly altered in both Hb and VTA, a subset showed concordance across regions, with upregulated DEGs showing enrichment for glutamatergic and GABAergic synaptic signaling and synapse organization, and downregulated DEGs enriched for lipid metabolism and ECM, which reflects shared neuroadaptation processes. However, nearly half of the shared DEGs (24 genes) displayed opposite regulation across the two regions, being upregulated in Hb but downregulated in VTA. These included genes involved in synaptic organization, neuronal signaling and cytoskeletal regulation (*Cttnbp2*, *Kcnq3*, *Kcnj10*,*Il1rap*, *Arhgap21*, *Rock1*, *Arap2*), metabolic and mitochondrial regulation (*Pnpla7*, *Slc25a27*, *Hif3a*, *Abcc5*), and protein modification or stress responses (*Usp31*, *Hltf*, *Htra1*). Given species differences and the distinct post-exposure time points across studies (24 h vs 90 min), these comparisons should be interpreted cautiously. Yet, this opposite transcriptional profile may suggest a region-specific action of fentanyl, with enhanced structural plasticity and metabolic demand in the Hb, contrasting with a dampening in the VTA. These molecular results are consistent with photometry recordings of the LHb during oral fentanyl self-administration [94], where progressive increases in drug-evoked LHb activity across three weeks of training suggest plasticity accompanying the transition from positive to negative reinforcement.

A study by Olusakin et al. [95] reported transcriptional adaptations across the mesolimbic pathway (VTA→NAc) following perinatal and juvenile fentanyl exposure in mice. Both VTA and NAc showed strong enrichment for genes linked to mitochondrial respiration, ECM remodeling, and synaptic and structural remodeling, functions that overlap with fentanyl-induced genes identified in our Hb and Amyg datasets. In the VTA, genes such as *Lrrtm2*, *Dynlt3*, and *Ndufaf4* were upregulated, reflecting enhanced synaptic and mitochondrial activity, whereas ECM-related genes (*Col9a3*, *Dact3*) and oxidative stress regulators (*Selenom*) were downregulated, indicating selective suppression of matrix and antioxidant processes. However, we did not observe significant overlap between our DEGs and those altered in the NAc in Olusakin and colleagues’ dataset. In addition, although no common DEGs were identified between our Hb and Amyg datasets and the mouse NAc profiles reported by Fox et al. [96], their data revealed mitochondrial, extracellular matrix, and synaptic GO term enrichments that match those dysregulated in our study. The absence of direct gene-level overlap likely reflects differences in species, brain regions, or experimental paradigms, with short oral fentanyl exposure and a long withdrawal period in their study, whereas our approach involved chronic fentanyl i.v self-administration and immediate DEGs assessment. Together, these findings support convergent, yet region-specific transcriptional adaptations in synaptic, metabolic, and structural processes across reward and aversion circuits following fentanyl exposure. These results are further confirmed by a recent study [44] where escalating fentanyl intake in long-access sessions was related to gene expression changes in calcium and potassium channels as well as synaptic signaling in the mPFC.

This transcriptional pattern extends beyond fentanyl self-administration and appears to be conserved across opioids and opiates. Indeed, morphine self-administration similarly enhanced synaptic, vesicular, and mitochondrial functions in the NAc while reducing extracellular matrix, vascular, and glial-related genes [40]. Likewise, heroin exposure produced comparable changes across several mesocorticolimbic regions, ventral hippocampus and BLA [9], reflecting a general shift toward heightened neuronal excitability and metabolic demand under diminished cellular support induced by mu receptor agonists.

To better understand which cell types are impacted by chronic fentanyl self-administration, we integrated our fentanyl DEGs with sn/scRNAseq data from rodent and human Hb and Amyg. This allowed us to overcome limitations in cell type heterogeneity in our bulk RNA-seq data, while leveraging the power of this approach to profile transcripts across different cell compartments, including the nucleus, cytoplasm, neuronal processes, and synapses. Consistent with previous sc/snRNA-seq studies that identified opioid-related transcriptional changes in glial populations in the rodent NAc and Amyg [30,34,40], we found enrichment of our fentanyl DEGs in oligodendrocytes, astrocytes and endothelial cells in both Hb and Amyg. These findings were conserved across species, and highlight that fentanyl impacts cell types critical for blood brain barrier (BBB) integrity and myelination. Notably, the BBB is an area of therapeutic interest as it regulates fentanyl access to the central nervous system [97], and opioids alter BBB homeostasis, structure, and integrity [98–100]. Further supporting a role for fentanyl in oligodendrocyte dysfunction, myelin pathology has been implicated in OUD [101,102] and chronic methadone treatment of primary rat glia cultures increases oligodendrocyte apoptosis and reduces myelinating capacity [103]. Future functional studies should investigate how fentanyl intake dysregulates the neurovascular unit.

Beyond glial cells, we also observed enrichment of upregulated and downregulated rat Hb fentanyl DEGs in specific mouse LHb cell types identified by Hashikawa et al. [51]. In particular, upregulated DEGs were enriched in mouse LHb.6, which is marked by expression of *Kcnmb4*, *Gpr151*, and *Chrm2*. These genes are also highly expressed in two *OPRM1*-expressing human Hb populations, LHb.2 and LHb.7 [52], suggesting possible conservation of fentanyl-sensitive mouse LHb.6 with human *OPRM1*-expressing LHb subpopulations. Notably, foot shock induces activity-dependent gene expression in LHb.6 [51], implicating this population in the processing of painful noxious stimuli and providing further support for the functional relevance of this LHb cell type in opioid signaling. Unfortunately, direct enrichment analyses of rat Hb fentanyl DEGs in our human Hb cell types did not yield any significant results, likely due to a lack of power resulting from the limited number of human Hb cells in our dataset.

In the Amyg, upregulated rat fentanyl DEGs were enriched in several excitatory and inhibitory populations, whereas downregulated DEGs were exclusively enriched in glial cell types, especially oligodendrocytes, which is consistent with findings in rodent and human Hb. We observed enrichment of upregulated rat fentanyl DEGs in multiple human fine level excitatory Amyg cell types, including *SATB2*+, *HGF*+, and *LAMP5*+ subclusters, which likely represent BLA excitatory populations. This is consistent with previous work showing that BLA glutamatergic principal neurons have increased intrinsic excitability and excitatory/inhibitory ratio in mice exposed to oral fentanyl consumption and withdrawal [24]. Future work should investigate the relationship between molecular and physiological changes in BLA pyramidal populations following chronic fentanyl intake and withdrawal. In terms of GABAergic neurons, vasoactive intestinal peptide (*VIP*)+ interneurons and *TSHZ1*+ intercalated neurons showed enrichment in upregulated genes in both rodents and humans. In the hippocampus, cortex, and striatum, there is mounting evidence for interactions between *VIP*+ interneurons and the opioid system, with *VIP*+ neurons showing *Oprm1* expression across many brain regions [104,105]. *TSHZ1*+ intercalated neurons are highly conserved across species [90], densely express *Oprm1,* and act to inhibit BLA and CeA circuitry under basal conditions [106,107]. However, *Oprm1* activation leads to inhibition of intercalated neurons, thus disinhibiting BLA and CeA reward circuitry underlying relapse of drug seeking behavior [108]. Together, these findings point towards a conserved opioid-driven disruption of glial support and inhibitory microcircuits that shifts amygdala function toward the disinhibition of neural circuits underlying relapse.

We acknowledge the present study has several limitations that set the stage for future investigations. First, we only perform molecular profiling at one timepoint ~90 minutes following the last self-administration session. We chose this time point to capture the transcriptomic changes following excessive chronic fentanyl use while avoiding transcriptomic changes induced by acute withdrawal. Hence, it will be important to distinguish the changes we observed from those following a single acute administration of fentanyl, although we expect distinct changes given prior comparisons of gene regulation after acute and chronic drug [9,109]. Additionally, it will be important to look at fentanyl-associated changes at other timepoints across different phases of opioid self-administration, including across acquisition, withdrawal and relapse. Given that the Hb is implicated in opioid withdrawal [110–112], future studies should assess transcriptomic changes following an abstinence period. Such changes could underlie alterations in risky decision making observed during fentanyl abstinence [113], and potentially contribute to relapse vulnerability. Second, while bulk RNA-seq is high throughput and cost-effective, higher resolution technologies such as snRNA-seq will be needed to validate inferred cell type-specific changes associated with chronic volitional fentanyl intake. As discussed above, snRNA-seq studies in the rodent NAc, VTA, and Amyg following opioid administration [34,40,43] have yielded valuable insights into neuronal and glial populations impacted by fentanyl and other opioids. Third, we acknowledge limitations in power that prevented the identification of DEGs associated with behavioral measures in fentanyl-administering rats, including total fentanyl intake. Future experiments with larger numbers of rats should leverage more complex behavioral analyses using tools, such as machine learning-based behavioral tracking [114], for integration with transcriptomic data. Furthermore, given previously reported sex differences in humans and rodents in the context of opioids [42,115–117], future studies should also include female rats as we were only able to assess males in the present study. Finally, it will also be important to consider different control groups, such as a natural reward group, to identify gene expression changes unique to an addictive drug like fentanyl from those induced by a non-addictive reward like sucrose.

In summary, we report transcriptional programs associated with fentanyl intake in the rat Hb and Amyg and link fentanyl-associated gene expression changes to specific cell types in the rodent and human brain. We provide evidence that genes altered by fentanyl in the rat Amyg overlap with genes implicated in genetic risk for neuropsychiatric disorders, including substance use disorders. Taken together, these findings highlight cell types and molecular pathways for further functional follow that could be targeted for potential therapeutic development.

## Supplementary Material

Supplement 1 **Table S1: Behavioral raw data per LgA self-administration session per rat.** Session-by-session raw behavioral data pertaining to operant (lever press) behaviors and infusions from each rat across all long-access self-administration sessions. See Table S3 for the description of these variables.
**Table S2: Behavioral rat data in LgA sessions.** Individual rat data for behavioral covariates relating to fentanyl intake and intake escalation across long-access self-administration sessions. See Table S3 for the description of these variables.
**Table S3: Dictionary of sample variables.** Description of sample/rat variables analyzed throughout the study. Related to Table S1, Table S2, and Table S4.
**Table S4: Sample metadata and QC metrics.** Sample level variables analyzed, including data regarding rat self-administration sessions and sample batches for RNA extraction, library preparation, and sequencing, as well as quality control metrics. See Table S3 for the description of these variables.
**Table S5: DEGs for substance in Hb.** Metadata, *limma* DE statistics, and Ensembl gene annotation for DEGs obtained for fentanyl vs. saline in Hb. See *limma* [65] documentation for these statistics definitions. Related to Figure 2, Table S8, Table S10.
**Table S6: DEGs for substance in Amyg.** Metadata, *limma* DE statistics, and Ensembl gene annotation for DEGs obtained for fentanyl vs. saline in Amyg. See *limma* [65] documentation for these statistics definitions. Related to Figure 2, Table S9, Table S10.
**Table S7. Common DEGs for substance in habenula and amygdala.** Metadata, region-specific *limma* DE statistics, and Ensembl gene annotation for overlapping DEGs for fentanyl vs. saline in habenula and amygdala. See *limma* [65] documentation for these statistics definitions. Related to Figure 2, Table S5, Table S6.
Table S8. Functional enrichment results for substance DEGs in Hb.
GO terms for biological processes (BP), molecular functions (MF), cellular components (CC), and KEGG pathways that are significantly enriched in up- and down-regulated DEGs for fentanyl vs. saline in Hb. Provided are the ID and description of each significant term/pathway, the number and fraction of up/down-regulated DEGs annotated to each term (Count and GeneRatio, respectively), as well as the list of such genes (geneID), the fraction of genes in universe annotated to each term (BgRatio), fold of enrichment, *p*-value, and FDR-corrected *p*-value. Related to Figure 2, Figure S10, Table S5.
**Table S9. Functional enrichment results for substance DEGs in Amyg.** Same as Table S8 but for up- and down-regulated DEGs for fentanyl vs. saline in Amyg. Related to Figure 2, Figure S10, Table S6.
**Table S10: Results for all DGE analyses and genes in Hb and Amyg.** Gene-level metadata and *limma* DE statistics of each gene for substance and rat behavior DGE analyses (fentanyl vs. saline, first hour infusion slope, total intake, and last session intake) in Hb and Amyg. See *limma* [65] documentation for these statistics definitions. Related to Figure 2. This table includes all the data from Table S5, Table S6, and Table S7.
**Table S11: Top 100 *MeanRatio* marker genes per cell type in mouse Hb.** For all cell subpopulations and Hb neuronal subpopulations in the Hb complex of control mice obtained in Hashikawa et al., 2020 [51], the top 100 most specific marker genes for each based on the MeanRatio method, are reported. See *DeconvoBuddies* [67] documentation for column description. Cell_type_resolution column corresponds to the resolution of the cell subpopulation for which the gene is a marker. Related to Figure 3.
**Table S12: Top 50 *MeanRatio* marker genes per cell type in human Hb.** For broad and fine cell types in the human Hb-enriched epithalamus of neurotypical control donors obtained in Yalcinbas et al., 2025 [52], the top 50 most specific marker genes for each based on the *MeanRatio* method, are reported. See *DeconvoBuddies* [67] documentation for column description. Cell_type_resolution column corresponds to the resolution of the cell type for which the gene is a marker. Related to Figure 3.
**Table S13: Top 100 *MeanRatio* marker genes per cell type in rat Amyg.** For main cell types and inhibitory neuronal subtypes in the Amyg of control rats obtained in Zhou et al., 2023 [54], the top 100 most specific marker genes for each based on the MeanRatio method, are reported. See *DeconvoBuddies* [67] documentation for column description. Cell_type_resolution column corresponds to the resolution of the cell type for which the gene is a marker. Related to Figure 4.
**Table S14: Top 100 *MeanRatio* marker genes per cell type in human Amyg.** For broad and fine cell types in the human Amyg of neurotypical control donors obtained in Yu et al., 2023 [53], the top 100 most specific marker genes for each based on the *MeanRatio* method, are reported. See *DeconvoBuddies* [67] documentation for column description. Cell_type_resolution column corresponds to the resolution of the cell type for which the gene is a marker. Related to Figure 4.

Supplement 2

## Data Availability

The source FASTQ files are publicly available from the NCBI Sequence Read Archive BioProject PRJNA1179901. All analysis code is available at https://github.com/LieberInstitute/fentanyl_rat_hb_amy.

## References

[1] Jones CM, Einstein EB, Compton WM. Changes in Synthetic Opioid Involvement in Drug Overdose Deaths in the United States, 2010–2016. JAMA. 2018;319:1819–1821. 29715347 10.1001/jama.2018.2844 PMC6583033

[2] Han Y, Yan W, Zheng Y, Khan MZ, Yuan K, Lu L. The rising crisis of illicit fentanyl use, overdose, and potential therapeutic strategies. Transl Psychiatry. 2019;9:282. 31712552 10.1038/s41398-019-0625-0 PMC6848196

[3] Bisaga A. What should clinicians do as fentanyl replaces heroin? Addiction. 2019;114:782–783. 30661265 10.1111/add.14522

[4] Kelly E, Sutcliffe K, Cavallo D, Ramos-Gonzalez N, Alhosan N, Henderson G. The anomalous pharmacology of fentanyl. Br J Pharmacol. 2023;180:797–812. 34030211 10.1111/bph.15573

[5] Meert TF, Vermeirsch HA. A preclinical comparison between different opioids: antinociceptive versus adverse effects. Pharmacol Biochem Behav. 2005;80:309–326. 15680184 10.1016/j.pbb.2004.12.002

[6] Torralva R, Eshleman AJ, Swanson TL, Schmachtenberg JL, Schutzer WE, Bloom SH, Fentanyl but not Morphine Interacts with Nonopioid Recombinant Human Neurotransmitter Receptors and Transporters. J Pharmacol Exp Ther. 2020;374:376–391. 32513839 10.1124/jpet.120.265561 PMC7430447

[7] Hill R, Santhakumar R, Dewey W, Kelly E, Henderson G. Fentanyl depression of respiration: Comparison with heroin and morphine. Br J Pharmacol. 2020;177:254–266. 31499594 10.1111/bph.14860 PMC6989952

[8] Welsch L, Bailly J, Darcq E, Kieffer BL. The negative affect of protracted opioid abstinence: progress and perspectives from rodent models. Biol Psychiatry. 2020;87:54–63. 31521334 10.1016/j.biopsych.2019.07.027 PMC6898775

[9] Browne CJ, Futamura R, Minier-Toribio A, Hicks EM, Ramakrishnan A, Martínez-Rivera FJ, Transcriptional signatures of heroin intake and relapse throughout the brain reward circuitry in male mice. Sci Adv. 2023;9:eadg8558. 37294757 10.1126/sciadv.adg8558 PMC10256172

[10] Koob GF, Volkow ND. Neurocircuitry of addiction. Neuropsychopharmacology. 2010;35:217–238. 19710631 10.1038/npp.2009.110 PMC2805560

[11] Namboodiri VMK, Rodriguez-Romaguera J, Stuber GD. The habenula. Curr Biol. 2016;26:R873–R877. 27728786 10.1016/j.cub.2016.08.051

[12] Velasquez KM, Molfese DL, Salas R. The role of the habenula in drug addiction. Front Hum Neurosci. 2014;8:174. 24734015 10.3389/fnhum.2014.00174 PMC3975120

[13] Lutz P-E, Kieffer BL. The multiple facets of opioid receptor function: implications for addiction. Curr Opin Neurobiol. 2013;23:473–479. 23453713 10.1016/j.conb.2013.02.005 PMC3702666

[14] Gardon O, Faget L, Chu Sin Chung P, Matifas A, Massotte D, Kieffer BL. Expression of mu opioid receptor in dorsal diencephalic conduction system: new insights for the medial habenula. Neuroscience. 2014;277:595–609. 25086313 10.1016/j.neuroscience.2014.07.053 PMC4164589

[15] Ding YQ, Kaneko T, Nomura S, Mizuno N. Immunohistochemical localization of mu-opioid receptors in the central nervous system of the rat. J Comp Neurol. 1996;367:375–402. 8698899 10.1002/(SICI)1096-9861(19960408)367:3<375::AID-CNE5>3.0.CO;2-2

[16] Bailly J, Allain F, Schwartz E, Tirel C, Dupuy C, Petit F, Habenular Neurons Expressing Mu Opioid Receptors Promote Negative Affect in a Projection-Specific Manner. Biol Psychiatry. 2023;93:1108–1117. 36496267 10.1016/j.biopsych.2022.09.013 PMC10027626

[17] Boulos LJ, Ben Hamida S, Bailly J, Maitra M, Ehrlich AT, Gavériaux-Ruff C, Mu opioid receptors in the medial habenula contribute to naloxone aversion. Neuropsychopharmacology. 2020;45:247–255. 31005059 10.1038/s41386-019-0395-7 PMC6901535

[18] Koob GF. Neurobiology of opioid addiction: opponent process, hyperkatifeia, and negative reinforcement. Biol Psychiatry. 2020;87:44–53. 31400808 10.1016/j.biopsych.2019.05.023

[19] Janak PH, Tye KM. From circuits to behaviour in the amygdala. Nature. 2015;517:284–292. 25592533 10.1038/nature14188 PMC4565157

[20] Hellemans KGC, Everitt BJ, Lee JLC. Disrupting reconsolidation of conditioned withdrawal memories in the basolateral amygdala reduces suppression of heroin seeking in rats. J Neurosci. 2006;26:12694–12699. 17151272 10.1523/JNEUROSCI.3101-06.2006 PMC6674846

[21] Wassum KM, Izquierdo A. The basolateral amygdala in reward learning and addiction. Neurosci Biobehav Rev. 2015;57:271–283. 26341938 10.1016/j.neubiorev.2015.08.017 PMC4681295

[22] Frenois F, Cador M, Caillé S, Stinus L, Le Moine C. Neural correlates of the motivational and somatic components of naloxone-precipitated morphine withdrawal. Eur J Neurosci. 2002;16:1377–1389. 12405997 10.1046/j.1460-9568.2002.02187.x

[23] Song J, Shao D, Guo X, Zhao Y, Cui D, Ma Q, Crucial role of feedback signals from prelimbic cortex to basolateral amygdala in the retrieval of morphine withdrawal memory. Sci Adv. 2019;5:eaat3210. 30801002 10.1126/sciadv.aat3210 PMC6382394

[24] Downs AM, Kmiec G, McElligott ZA. Oral fentanyl consumption and withdrawal impairs fear extinction learning and enhances basolateral amygdala principal neuron excitatory-inhibitory balance in male and female mice. Addiction Neuroscience. 2024;13:100182. 39742087 10.1016/j.addicn.2024.100182 PMC11687336

[25] Lüscher C, Janak PH. Consolidating the circuit model for addiction. Annu Rev Neurosci. 2021;44:173–195. 33667115 10.1146/annurev-neuro-092920-123905

[26] Watanabe T, Yamamoto R, Maeda A, Nakagawa T, Minami M, Satoh M. Effects of excitotoxic lesions of the central or basolateral nucleus of the amygdala on naloxone-precipitated withdrawal-induced conditioned place aversion in morphine-dependent rats. Brain Res. 2002;958:423–428. 12470879 10.1016/s0006-8993(02)03468-6

[27] Chaudun F, Python L, Liu Y, Hiver A, Cand J, Kieffer BL, Distinct μ-opioid ensembles trigger positive and negative fentanyl reinforcement. Nature. 2024;630:141–148. 38778097 10.1038/s41586-024-07440-x PMC11153127

[28] Li Z, Li C, Yin P, Wang ZJ, Luo F. Inhibition of CaMKIIα in the Central Nucleus of Amygdala Attenuates Fentanyl-Induced Hyperalgesia in Rats. J Pharmacol Exp Ther. 2016;359:82–89. 27451410 10.1124/jpet.116.233817

[29] Bai T, Chen H, Hu W, Liu J, Lin X, Chen S, Amygdala Metabotropic Glutamate Receptor 1 Influences Synaptic Transmission to Participate in Fentanyl-Induced Hyperalgesia in Rats. Cell Mol Neurobiol. 2023;43:1401–1412. 35798932 10.1007/s10571-022-01248-x PMC11414450

[30] Avey D, Sankararaman S, Yim AKY, Barve R, Milbrandt J, Mitra RD. Single-Cell RNA-Seq Uncovers a Robust Transcriptional Response to Morphine by Glia. Cell Rep. 2018;24:3619–3629.e4. 30257220 10.1016/j.celrep.2018.08.080 PMC6357782

[31] Loguinov AV, Anderson LM, Crosby GJ, Yukhananov RY. Gene expression following acute morphine administration. Physiol Genomics. 2001;6:169–181. 11526201 10.1152/physiolgenomics.2001.6.3.169

[32] Korostynski M, Piechota M, Kaminska D, Solecki W, Przewlocki R. Morphine effects on striatal transcriptome in mice. Genome Biol. 2007;8:R128. 17598886 10.1186/gb-2007-8-6-r128 PMC2394777

[33] Liu SX, Gades MS, Swain Y, Ramakrishnan A, Harris AC, Tran PV, Repeated morphine exposure activates synaptogenesis and other neuroplasticity-related gene networks in the dorsomedial prefrontal cortex of male and female rats. Drug Alcohol Depend. 2021;221:108598. 33626484 10.1016/j.drugalcdep.2021.108598 PMC8026706

[34] Yan Y, Truitt B, Tao J, Boyles SM, Antoine D, Hulme W, Single-cell profiling of glial cells from the mouse amygdala under opioid dependent and withdrawal states. IScience. 2023;26:108166. 37915593 10.1016/j.isci.2023.108166 PMC10616319

[35] Zhang Y, Liang Y, Levran O, Randesi M, Yuferov V, Zhao C, Alterations of expression of inflammation/immune-related genes in the dorsal and ventral striatum of adult C57BL/6J mice following chronic oxycodone self-administration: a RNA sequencing study. Psychopharmacology (Berl). 2017;234:2259–2275. 28653080 10.1007/s00213-017-4657-y PMC5826641

[36] Zhang Y, Liang Y, Randesi M, Yuferov V, Zhao C, Kreek MJ. Chronic Oxycodone Self-administration Altered Reward-related Genes in the Ventral and Dorsal Striatum of C57BL/6J Mice: An RNA-seq Analysis. Neuroscience. 2018;393:333–349. 30059705 10.1016/j.neuroscience.2018.07.032

[37] Yuferov V, Zhang Y, Liang Y, Zhao C, Randesi M, Kreek MJ. Oxycodone Self-Administration Induces Alterations in Expression of Integrin, Semaphorin and Ephrin Genes in the Mouse Striatum. Front Psychiatry. 2018;9:257. 29946272 10.3389/fpsyt.2018.00257 PMC6005861

[38] Vassoler FM, Budge KE, Isgate SB, Gildawie KR, Byrnes EM. Neuroplasticity-related genes correlate with individual differences in distinct phases of oxycodone self-administration in male rats. Neuropharmacology. 2024;254:109972. 38710443 10.1016/j.neuropharm.2024.109972 PMC11164234

[39] Blackwood CA, McCoy MT, Ladenheim B, Cadet JL. Escalated Oxycodone Self-Administration and Punishment: Differential Expression of Opioid Receptors and Immediate Early Genes in the Rat Dorsal Striatum and Prefrontal Cortex. Front Neurosci. 2019;13:1392. 31998063 10.3389/fnins.2019.01392 PMC6962106

[40] Reiner BC, Zhang Y, Stein LM, Perea ED, Arauco-Shapiro G, Ben Nathan J, Single nucleus transcriptomic analysis of rat nucleus accumbens reveals cell type-specific patterns of gene expression associated with volitional morphine intake. Transl Psychiatry. 2022;12:374. 36075888 10.1038/s41398-022-02135-1 PMC9458645

[41] Cannella N, Tambalo S, Lunerti V, Scuppa G, de Vivo L, Abdulmalek S, Long-access heroin self-administration induces region specific reduction of grey matter volume and microglia reactivity in the rat. Brain Behav Immun. 2024;118:210–220. 38452987 10.1016/j.bbi.2024.03.003

[42] Townsend EA, Kim RK, Robinson HL, Marsh SA, Banks ML, Hamilton PJ. Opioid withdrawal produces sex-specific effects on fentanyl-vs.-food choice and mesolimbic transcription. Biological Psychiatry Global Open Science. 2021;1:112–122. 34458885 10.1016/j.bpsgos.2021.04.009 PMC8389189

[43] Fox ME, Montemarano A, Ostman AE, Basu M, Herb B, Ament SA, Transcriptional signatures of fentanyl use in the mouse ventral tegmental area. Addict Biol. 2024;29:e13403. 38735880 10.1111/adb.13403 PMC11089014

[44] Keady J, Charnigo R, Shaykin JD, Prantzalos ER, Xia M, Denehy E, Behavioral and genetic markers of susceptibility to escalate fentanyl intake. BioRxiv. 2025. January 29, 2025. 10.1101/2024.12.06.627259. PMC13234145 42020354

[45] Seney ML, Kim S-M, Glausier JR, Hildebrand MA, Xue X, Zong W, Transcriptional alterations in dorsolateral prefrontal cortex and nucleus accumbens implicate neuroinflammation and synaptic remodeling in opioid use disorder. Biol Psychiatry. 2021;90:550–562. 34380600 10.1016/j.biopsych.2021.06.007 PMC8463497

[46] Phan BN, Ray MH, Xue X, Fu C, Fenster RJ, Kohut SJ, Single nuclei transcriptomics in human and non-human primate striatum in opioid use disorder. Nat Commun. 2024;15:878. 38296993 10.1038/s41467-024-45165-7 PMC10831093

[47] Ellis MS, Kasper ZA, Cicero TJ. Twin epidemics: The surging rise of methamphetamine use in chronic opioid users. Drug Alcohol Depend. 2018;193:14–20. 30326396 10.1016/j.drugalcdep.2018.08.029

[48] Cicero TJ, Ellis MS, Kasper ZA. Polysubstance use: A broader understanding of substance use during the opioid crisis. Am J Public Health. 2020;110:244–250. 31855487 10.2105/AJPH.2019.305412 PMC6951387

[49] Compton WM, Valentino RJ, DuPont RL. Polysubstance use in the U.S. opioid crisis. Mol Psychiatry. 2021;26:41–50. 33188253 10.1038/s41380-020-00949-3 PMC7815508

[50] Ho M-F, Zhang C, Moon I, Zhu X, Coombes BJ, Biernacka J, Single cell transcriptomics reveals distinct transcriptional responses to oxycodone and buprenorphine by iPSC-derived brain organoids from patients with opioid use disorder. Mol Psychiatry. 2024;29:1636–1646. 36302966 10.1038/s41380-022-01837-8 PMC10588459

[51] Hashikawa Y, Hashikawa K, Rossi MA, Basiri ML, Liu Y, Johnston NL, Transcriptional and spatial resolution of cell types in the mammalian habenula. Neuron. 2020;106:743–758.e5. 32272058 10.1016/j.neuron.2020.03.011 PMC7285796

[52] Yalcinbas EA, Ajanaku B, Nelson ED, Garcia-Flores R, Eagles NJ, Montgomery KD, Transcriptomic analysis of the human habenula in schizophrenia. Am J Psychiatry. 2025;182:991–1006. 41174894 10.1176/appi.ajp.20240776 PMC13008474

[53] Yu B, Zhang Q, Lin L, Zhou X, Ma W, Wen S, Molecular and cellular evolution of the amygdala across species analyzed by single-nucleus transcriptome profiling. Cell Discov. 2023;9:19. 36788214 10.1038/s41421-022-00506-y PMC9929086

[54] Zhou JL, de Guglielmo G, Ho AJ, Kallupi M, Pokhrel N, Li H-R, Single-nucleus genomics in outbred rats with divergent cocaine addiction-like behaviors reveals changes in amygdala GABAergic inhibition. Nat Neurosci. 2023;26:1868–1879. 37798411 10.1038/s41593-023-01452-y PMC10620093

[55] Hatoum AS, Colbert SMC, Johnson EC, Huggett SB, Deak JD, Pathak G, Multivariate genome-wide association meta-analysis of over 1 million subjects identifies loci underlying multiple substance use disorders. Nat Ment Health. 2023;1:210–223. 37250466 10.1038/s44220-023-00034-y PMC10217792

[56] Deak JD, Zhou H, Galimberti M, Levey DF, Wendt FR, Sanchez-Roige S, Genome-wide association study in individuals of European and African ancestry and multi-trait analysis of opioid use disorder identifies 19 independent genome-wide significant risk loci. Mol Psychiatry. 2022;27:3970–3979. 35879402 10.1038/s41380-022-01709-1 PMC9718667

[57] Levey DF, Stein MB, Wendt FR, Pathak GA, Zhou H, Aslan M, Bi-ancestral depression GWAS in the Million Veteran Program and meta-analysis in >1.2 million individuals highlight new therapeutic directions. Nat Neurosci. 2021;24:954–963. 34045744 10.1038/s41593-021-00860-2 PMC8404304

[58] Howard DM, Adams MJ, Clarke T-K, Hafferty JD, Gibson J, Shirali M, Genome-wide meta-analysis of depression identifies 102 independent variants and highlights the importance of the prefrontal brain regions. Nat Neurosci. 2019;22:343–352. 30718901 10.1038/s41593-018-0326-7 PMC6522363

[59] Forstner AJ, Awasthi S, Wolf C, Maron E, Erhardt A, Czamara D, Genome-wide association study of panic disorder reveals genetic overlap with neuroticism and depression. Mol Psychiatry. 2021;26:4179–4190. 31712720 10.1038/s41380-019-0590-2

[60] Trubetskoy V, Pardiñas AF, Qi T, Panagiotaropoulou G, Awasthi S, Bigdeli TB, Mapping genomic loci implicates genes and synaptic biology in schizophrenia. Nature. 2022;604:502–508. 35396580 10.1038/s41586-022-04434-5 PMC9392466

[61] Thomsen M, Caine SB. Chronic intravenous drug self-administration in rats and mice. Curr Protoc Neurosci. 2005;Chapter 9:Unit 9.20. 10.1002/0471142301.ns0920s32 18428629

[62] Fragale JE, James MH, Aston-Jones G. Intermittent self-administration of fentanyl induces a multifaceted addiction state associated with persistent changes in the orexin system. Addict Biol. 2021;26:e12946. 32798290 10.1111/adb.12946 PMC7882007

[63] Eagles NJ, Burke EE, Leonard J, Barry BK, Stolz JM, Huuki L, SPEAQeasy: a scalable pipeline for expression analysis and quantification for R/bioconductor-powered RNA-seq analyses. BMC Bioinformatics. 2021;22:224. 33932985 10.1186/s12859-021-04142-3 PMC8088074

[64] Robinson MD, Oshlack A. A scaling normalization method for differential expression analysis of RNA-seq data. Genome Biol. 2010;11:R25. 20196867 10.1186/gb-2010-11-3-r25 PMC2864565

[65] Ritchie ME, Phipson B, Wu D, Hu Y, Law CW, Shi W, limma powers differential expression analyses for RNA-sequencing and microarray studies. Nucleic Acids Res. 2015;43:e47. 25605792 10.1093/nar/gkv007 PMC4402510

[66] Yu G, Wang L-G, Han Y, He Q-Y. clusterProfiler: an R package for comparing biological themes among gene clusters. OMICS. 2012;16:284–287. 22455463 10.1089/omi.2011.0118 PMC3339379

[67] Huuki-Myers LA, Montgomery KD, Kwon SH, Cinquemani S, Eagles NJ, Gonzalez-Padilla D, Benchmark of cellular deconvolution methods using a multi-assay dataset from postmortem human prefrontal cortex. Genome Biol. 2025;26:88. 40197307 10.1186/s13059-025-03552-3 PMC11978107

[68] Durinck S, Moreau Y, Kasprzyk A, Davis S, De Moor B, Brazma A, BioMart and Bioconductor: a powerful link between biological databases and microarray data analysis. Bioinformatics. 2005;21:3439–3440. 16082012 10.1093/bioinformatics/bti525

[69] de Leeuw CA, Mooij JM, Heskes T, Posthuma D. MAGMA: generalized gene-set analysis of GWAS data. PLoS Comput Biol. 2015;11:e1004219. 25885710 10.1371/journal.pcbi.1004219 PMC4401657

[70] 1000 Genomes Project Consortium, Auton A, Brooks LD, Durbin RM, Garrison EP, Kang HM, A global reference for human genetic variation. Nature. 2015;526:68–74. 26432245 10.1038/nature15393 PMC4750478

[71] Gonzalez-Padilla D, Eagles N, Collado-Torres L. LieberInstitute/fentanyl_rat_hb_amy. Zenodo. 2025. November 10, 2025. 10.5281/zenodo.17573970.

[72] Team RC. R: A Language and Environment for Statistical Computing. Vienna, Austria: CRAN; 2025.

[73] Huber W, Carey VJ, Gentleman R, Anders S, Carlson M, Carvalho BS, Orchestrating high-throughput genomic analysis with Bioconductor. Nat Methods. 2015;12:115–121. 25633503 10.1038/nmeth.3252 PMC4509590

[74] Wickham H. ggplot2: Elegant Graphics for Data Analysis (Use R!). 2nd ed. Cham: Springer; 2016.

[75] Smith KR, Jones KA, Kopeikina KJ, Burette AC, Copits BA, Yoon S, Cadherin-10 Maintains Excitatory/Inhibitory Ratio through Interactions with Synaptic Proteins. J Neurosci. 2017;37:11127–11139. 29030434 10.1523/JNEUROSCI.1153-17.2017 PMC5688522

[76] Shih P-Y, Hsieh B-Y, Lin M-H, Huang T-N, Tsai C-Y, Pong W-L, CTTNBP2 Controls Synaptic Expression of Zinc-Related Autism-Associated Proteins and Regulates Synapse Formation and Autism-like Behaviors. Cell Rep. 2020;31:107700. 32492416 10.1016/j.celrep.2020.107700

[77] El Ghaleb Y, Schneeberger PE, Fernández-Quintero ML, Geisler SM, Pelizzari S, Polstra AM, CACNA1I gain-of-function mutations differentially affect channel gating and cause neurodevelopmental disorders. Brain. 2021;144:2092–2106. 33704440 10.1093/brain/awab101 PMC8422349

[78] Wilson SH, Bailey AM, Nourse CR, Mattei MG, Byrne JA. Identification of MAL2, a novel member of the mal proteolipid family, though interactions with TPD52-like proteins in the yeast two-hybrid system. Genomics. 2001;76:81–88. 11549320 10.1006/geno.2001.6610

[79] Funakoshi A, Honda T, Ito T, Tokura Y. Cholecystokinin receptor antagonist suppresses melanoma growth by inducing apoptosis of tumor cells. JID Innov. 2022;2:100153. 36262666 10.1016/j.xjidi.2022.100153 PMC9573926

[80] Ludtmann MHR, Angelova PR, Horrocks MH, Choi ML, Rodrigues M, Baev AY, α-synuclein oligomers interact with ATP synthase and open the permeability transition pore in Parkinson’s disease. Nat Commun. 2018;9:2293. 29895861 10.1038/s41467-018-04422-2 PMC5997668

[81] Bromley-Coolidge S, Iruegas D, Appel B. Cspg4 sculpts oligodendrocyte precursor cell morphology. Differentiation. 2024;140:100819. 39566199 10.1016/j.diff.2024.100819 PMC11637897

[82] Goto S, Kawakatsu M, Izumi S-I, Urata Y, Kageyama K, Ihara Y, Glutathione S-transferase pi localizes in mitochondria and protects against oxidative stress. Free Radic Biol Med. 2009;46:1392–1403. 19269317 10.1016/j.freeradbiomed.2009.02.025

[83] Kamada F, Aoki Y, Narisawa A, Abe Y, Komatsuzaki S, Kikuchi A, A genome-wide association study identifies RNF213 as the first Moyamoya disease gene. J Hum Genet. 2011;56:34–40. 21048783 10.1038/jhg.2010.132

[84] Panda S, Setia M, Kaur N, Shepal V, Arora V, Singh DK, Noncoding RNA Ginir functions as an oncogene by associating with centrosomal proteins. PLoS Biol. 2018;16:e2004204. 30296263 10.1371/journal.pbio.2004204 PMC6193740

[85] Phillips MC. Is ABCA1 a lipid transfer protein? J Lipid Res. 2018;59:749–763. 29305383 10.1194/jlr.R082313 PMC5928442

[86] Macian F. NFAT proteins: key regulators of T-cell development and function. Nat Rev Immunol. 2005;5:472–484. 15928679 10.1038/nri1632

[87] Steffensen LB, Stubbe J, Lindholt JS, Beck HC, Overgaard M, Bloksgaard M, Basement membrane collagen IV deficiency promotes abdominal aortic aneurysm formation. Sci Rep. 2021;11:12903. 34145342 10.1038/s41598-021-92303-y PMC8213747

[88] Jeremic D, Sanchez-Rodriguez I, Jimenez-Diaz L, Navarro-Lopez JD. Therapeutic potential of targeting G protein-gated inwardly rectifying potassium (GIRK) channels in the central nervous system. Pharmacol Ther. 2021;223:107808. 33476640 10.1016/j.pharmthera.2021.107808

[89] Aksoy-Aksel A, Gall A, Seewald A, Ferraguti F, Ehrlich I. Midbrain dopaminergic inputs gate amygdala intercalated cell clusters by distinct and cooperative mechanisms in male mice. ELife. 2021;10. 10.7554/eLife.63708 PMC8143799 34028352

[90] Totty MS, Cervera Juanes R, Bach SV, Ben Ameur L, Valentine MR, Simons E, Transcriptomic diversity of amygdalar subdivisions across humans and nonhuman primates. Sci Adv. 2025;11:eadw1029. 40961182 10.1126/sciadv.adw1029 PMC12442850

[91] Wood CP, Shin Y, Balaguer MG, Campo P, Dirik S, Montoya BA, Perinatal fentanyl exposure drives enduring addiction risk and central amygdala gene dysregulation. Neuropharmacology. 2025;278:110581. 40582636 10.1016/j.neuropharm.2025.110581 PMC12377289

[92] Uchegbu C, Nguyen T, Sood I, Blankenship K, He H, Stanwood GD, Prenatal fentanyl exposure affects social dominance and myelination patterns in the adult mouse brain. Neuropharmacology. 2025;278:110583. 40592379 10.1016/j.neuropharm.2025.110583 PMC13430950

[93] Malone SG, Tavakoli NS, Keller PS, Bardo MT, Ortinski PI. Calcium imaging of central amygdala activity after escalation of fentanyl self-administration. Neuropharmacology. 2025;271:110370. 39988277 10.1016/j.neuropharm.2025.110370 PMC11930605

[94] Coffey KR, Nickelson WB, Dawkins AJ, Neumaier JF. Rapid appearance of negative emotion during oral fentanyl self-administration in male and female rats. Addict Biol. 2023;28:e13344. 38017643 10.1111/adb.13344 PMC10745948

[95] Olusakin J, Kumar G, Basu M, Calarco CA, Fox ME, Alipio JB, Transcriptomic profiling of reward and sensory brain areas in perinatal fentanyl exposed juvenile mice. Neuropsychopharmacology. 2023;48:1724–1734. 37400565 10.1038/s41386-023-01639-8 PMC10579237

[96] Fox ME, Wulff AB, Franco D, Choi EY, Calarco CA, Engeln M, Adaptations in nucleus accumbens neuron subtypes mediate negative affective behaviors in fentanyl abstinence. Biol Psychiatry. 2023;93:489–501. 36435669 10.1016/j.biopsych.2022.08.023 PMC9931633

[97] Schaefer CP, Tome ME, Davis TP. The opioid epidemic: a central role for the blood brain barrier in opioid analgesia and abuse. Fluids Barriers CNS. 2017;14:32. 29183383 10.1186/s12987-017-0080-3 PMC5706290

[98] Reymond S, Vujić T, Schvartz D, Sanchez J-C. Morphine-induced modulation of Nrf2-antioxidant response element signaling pathway in primary human brain microvascular endothelial cells. Sci Rep. 2022;12:4588. 35301408 10.1038/s41598-022-08712-0 PMC8931063

[99] Sun E, Torices S, Osborne OM, Toborek M. Microvascular dysfunction, mitochondrial reprogramming, and inflammasome activation as critical regulators of ischemic stroke severity induced by chronic exposure to prescription opioids. J Neurosci. 2025;45. 10.1523/JNEUROSCI.0614-24.2024 PMC11841762 39753298

[100] Pimentel E, Sivalingam K, Doke M, Samikkannu T. Effects of Drugs of Abuse on the Blood-Brain Barrier: A Brief Overview. Front Neurosci. 2020;14:513. 32670001 10.3389/fnins.2020.00513 PMC7326150

[101] Bora E, Yücel M, Fornito A, Pantelis C, Harrison BJ, Cocchi L, White matter microstructure in opiate addiction. Addict Biol. 2012;17:141–148. 21070508 10.1111/j.1369-1600.2010.00266.x

[102] Liu H, Li L, Hao Y, Cao D, Xu L, Rohrbaugh R, Disrupted white matter integrity in heroin dependence: a controlled study utilizing diffusion tensor imaging. Am J Drug Alcohol Abuse. 2008;34:562–575. 18720268 10.1080/00952990802295238

[103] De Gregorio C, Gallardo J, Berríos-Cárcamo P, Handy Á, Santapau D, González-Madrid A, Methadone directly impairs central nervous system cells in vitro. Sci Rep. 2024;14:16978. 39043899 10.1038/s41598-024-67860-7 PMC11266518

[104] Hosseinzadeh Sahafi O, Sardari M, Alijanpour S, Rezayof A. Shared mechanisms of gabaergic and opioidergic transmission regulate corticolimbic reward systems and cognitive aspects of motivational behaviors. Brain Sci. 2023;13. 37239287 10.3390/brainsci13050815 PMC10216078

[105] Zamfir M, Sharif B, Locke S, Ehrlich AT, Ochandarena NE, Scherrer G, Distinct and sex-specific expression of mu opioid receptors in anterior cingulate and somatosensory S1 cortical areas. Pain. 2023;164:703–716. 35973045 10.1097/j.pain.0000000000002751 PMC10026835

[106] Aksoy-Aksel A, Ferraguti F, Holmes A, Lüthi A, Ehrlich I. Amygdala intercalated cells form an evolutionarily conserved system orchestrating brain networks. Nat Neurosci. 2025;28:234–247. 39672964 10.1038/s41593-024-01836-8 PMC12318446

[107] Blaesse P, Goedecke L, Bazelot M, Capogna M, Pape H-C, Jüngling K. μ-Opioid Receptor-Mediated Inhibition of Intercalated Neurons and Effect on Synaptic Transmission to the Central Amygdala. J Neurosci. 2015;35:7317–7325. 25972162 10.1523/JNEUROSCI.0204-15.2015 PMC4429148

[108] Gregoriou GC, Patel SD, Pyne S, Winters BL, Bagley EE. Opioid withdrawal abruptly disrupts amygdala circuit function by reducing peptide actions. J Neurosci. 2023;43:1668–1681. 36781220 10.1523/JNEUROSCI.1317-22.2022 PMC10010477

[109] Phillips RA, Tuscher JJ, Fitzgerald ND, Wan E, Zipperly ME, Duke CG, Distinct subpopulations of D1 medium spiny neurons exhibit unique transcriptional responsiveness to cocaine. Mol Cell Neurosci. 2023;125:103849. 36965548 10.1016/j.mcn.2023.103849 PMC10898607

[110] Valentinova K, Tchenio A, Trusel M, Clerke JA, Lalive AL, Tzanoulinou S, Morphine withdrawal recruits lateral habenula cytokine signaling to reduce synaptic excitation and sociability. Nat Neurosci. 2019;22:1053–1056. 31209376 10.1038/s41593-019-0421-4

[111] Meye FJ, Trusel M, Soiza-Reilly M, Mameli M. Neural circuit adaptations during drug withdrawal - Spotlight on the lateral habenula. Pharmacol Biochem Behav. 2017;162:87–93. 28843423 10.1016/j.pbb.2017.08.007

[112] Wang J, Li M, Wang P, Zha Y, He Z, Li Z. Inhibition of the lateral habenular CaMKII abolishes naloxone-precipitated conditioned place aversion in morphine-dependent mice. Neurosci Lett. 2017;653:64–70. 28527717 10.1016/j.neulet.2017.05.027

[113] Hrelja KM, Kawkab C, Avramidis DK, Ramaiah S, Winstanley CA. Increased risky choice during forced abstinence from fentanyl on the cued rat gambling task. Psychopharmacology (Berl). 2025;242:173–187. 39078498 10.1007/s00213-024-06659-w

[114] Mathis A, Mamidanna P, Cury KM, Abe T, Murthy VN, Mathis MW, DeepLabCut: markerless pose estimation of user-defined body parts with deep learning. Nat Neurosci. 2018;21:1281–1289. 30127430 10.1038/s41593-018-0209-y

[115] Towers EB, Setaro B, Lynch WJ. Sex- and Dose-Dependent Differences in the Development of an Addiction-Like Phenotype Following Extended-Access Fentanyl Self-Administration. Front Pharmacol. 2022;13:841873. 35370634 10.3389/fphar.2022.841873 PMC8968863

[116] Gaulden AD, Burson N, Sadik N, Ghosh I, Khan SJ, Brummelte S, Effects of fentanyl on acute locomotor activity, behavioral sensitization, and contextual reward in female and male rats. Drug Alcohol Depend. 2021;229:109101. 34628096 10.1016/j.drugalcdep.2021.109101 PMC8671359

[117] Knouse MC, Briand LA. Behavioral sex differences in cocaine and opioid use disorders: The role of gonadal hormones. Neurosci Biobehav Rev. 2021;128:358–366. 34214512 10.1016/j.neubiorev.2021.06.038 PMC8363946

