# Supplementary material for: Transcriptional response to chronic long-access fentanyl self-administration in rat habenula and amygdala
Source: bioRxiv. 2025 Dec 12:2025.11.25.690517. Originally published 2025 Nov 28. Preprint. [Version 2] doi: 10.1101/2025.11.25.690517 (PMC12699572; doi:10.1101/2025.11.25.690517)
Supplement: Supplement 2 [file NIHPP2025.11.25.690517v2-supplement-2.pdf]

bioRxiv preprint doi: https://doi.org/10.1101/2025.11.25.690517; this version posted December 12, 2025. The copyright holder for this preprint (which was not certified by peer review) is the author/funder, who has granted bioRxiv a license to display the preprint in perpetuity. It is made available under aCC-BY 4.0 International license.

Supplementary Methods

Library construction and bulk RNA sequencing (RNA-seq)

Total RNA was extracted from habenula and amygdala tissue samples using the Qiagen RNeasy Micro Kit (Cat. No.: 74004). Paired-end strand-specific sequencing libraries were prepared for 33 samples (16 habenula, 17 amygdala) from 100 ng total RNA input for amygdala samples, and 10 ng total RNA input from low-yield habenula samples. For amygdala samples, TruSeq Stranded Total RNA Library Preparation kit with Ribo-Zero H/M/R Gold ribosomal RNA depletion was used (min. 0.5 ug of total RNA needed per sample). For low-yield habenula samples, Illumina Stranded Total RNA Prep Library Preparation kit with Ribo-Zero Plus was used (~1 ng to 1000 ng of total RNA needed per sample). For quality control, synthetic External RNA Controls Consortium (ERCC) RNA Mix 1 (Thermo Fisher Scientific) was spiked into each sample. Libraries were sequenced on an Illumina NovaSeq 6000 S4 (152 bp PE) producing ~80 million (median 102,396,054, mean 98,533,714) 152 bp paired-end reads per sample.

RNA-seq data processing

Gene expression quantification

SPEAQeasy pipeline version 87ba0b4 [63], a *Nextflow* v20.01.0 [118] workflow for *HISAT2* v2.2.1 [119], was used with default settings to assess the quality of the sequencing reads and quantify the gene expression in the samples using the rat genome assembly mRatBN7.2 from Ensembl release 109 [120,121]. A *RangedSummarizedExperiment* R object [73] with gene counts for 30,452 genes across 33 samples was built by *SPEAQeasy*; this object included sample quality metrics that were used for exploratory analyses (**Supplementary Methods: Exploratory Data Analysis**).

Filtering of lowly-expressed genes

Lowly-expressed genes were filtered using `filterByExpr()` from *edgeR* v3.43.7 [122] in which only genes with at least 15 total reads across all samples and with 10 or more counts in at least *n* samples are retained, where *n* is defined as 70% the size of the smallest sample group. After this step, 16,708 genes (54.86%) were retained for downstream analyses.

Count normalization

Raw expression counts of the genes in the 33 samples were normalized by trimmed mean of M-values (TMM) [64] using `calcNormFactors()` from *edgeR* v3.43.7 [122] to compute normalization factors for library size rescaling. *edgeR* `cpm()` [122] was subsequently used to obtain counts per million (CPM) in a logarithmic scale: approximately $\log_2$(CPM+0.5).

Exploratory Data Analysis (EDA)

Sample Quality Control Analysis (QCA)

Sample-level gene-based quality control (QC) metrics computed by *SPEAQeasy* [63] on raw counts before gene filtering and count normalization steps (**Table S3**, **Table S4**), were compared across the different brain regions, substances, preparation batches, and rat administration sessions. Hb samples presented lower yields of RNA compared to Amyg samples, which in turn decreased their library sizes, number of detected genes, and read mapping rates (**Figure S2**). The third RNA extraction batch was performed on additional samples and

bioRxiv preprint doi: https://doi.org/10.1101/2025.11.25.690517; this version posted December 12, 2025. The copyright holder for this preprint (which was not certified by peer review) is the author/funder, who has granted bioRxiv a license to display the preprint in perpetuity. It is made available under aCC-BY 4.0 International license.

resulted in more comparable yields with the second higher-yield Amyg batch. Hb and Amyg samples from the third RNA extraction batch presented good RNA amounts and library sizes, and higher mapping rates than their counterparts in the first and second batches, respectively (**Figure S3**). No association was observed between sample quality metrics and total number of fentanyl self-administration sessions (**Figure S4**). Hb and Amyg samples were analyzed separately in subsequent steps.

Low-quality samples were defined through the identification of outlier QC metrics with `isOutlier()` from *scater* v1.30.1 [123], which takes as outliers those values that are 3 median-absolute-deviations (MAD) away from the median. For Hb, one of the 16 samples was detected as an outlier for the number of detected genes (**Figure S5A**) and for Amyg, 3 of the 17 samples were detected as outliers for mitochondrial mapping rate, concordant mapping rate, or number of detected genes (**Figure S5B**); two of the three Amyg outliers were from the third RNA extraction batch. However, these four samples had no other outlier QC metrics (**Figure S5**) and were not removed but examined further in principal component (PC) plots (**Supplementary Methods: Sample-level gene expression variation and manual QC inspection**). Sample QC metrics are described in **Table S3**.

Sample-level gene expression variation and manual QC inspection

Sources of gene expression variation between samples were explored with Principal Component Analysis (PCA) on log-normalized counts of expressed genes. RNA extraction batch appeared as a major driver of gene expression variability in both Hb and Amyg (**Figure S6**), and substance had a greater contribution among Amyg samples (**Figure S6B**). The four previous outlier samples in QC metrics (**Figure S5**) were not outliers on PC plots, although other samples were detected as outliers by PCA (**Figure S7**). Both QC metrics and PCA outlier samples were subjected to manual inspection of all their QC metrics, finding only attenuated differences in the quality of these samples compared to the non-outlier ones, as well as high-quality metrics for these samples relative to the global metrics estimates (**Figure S7**). All Hb and Amyg samples were retained for posterior analyses.

Gene-level expression variation and covariate selection for DGE

To explore the contributions of sample-level variables on gene expression variation and guide variable selection to model gene expression for DGE analysis, we first computed the percentage of variance of gene expression explained by each covariate individually with `getVarianceExplained()` from *scater* v1.30.1 [123]. We implemented this analysis taking all Hb and Amyg samples separately (**Figure S8**) and then subsetting to fentanyl-administered samples from each brain region (**Figure S8**), as additional DGE analyses were performed on fentanyl-administered samples only (see further below and **Supplementary Methods: Differential Gene Expression analysis**).

Second, for the same sample groups, we performed pairwise Canonical Correlation Analysis (CCA) with `canCorPairs()` of *variancePartition* v1.32.5 [124] to identify pairs of correlated variables (**Figure S8**). To remove redundant and minority contributing variables and to avoid unmasking true drivers of variation, models for DGE between fentanyl vs. saline administration and for rat behavior in Hb and Amyg were defined by discarding:

i) variables highly correlated with “substance” (fentanyl vs. saline; **Figure S8**) or “behavioral covariates” (i.e. total fentanyl intake, last session fentanyl intake, or slope for the rats’ fentanyl intake in the first hour of each session; **Figure S8**);
ii) variables highly correlated with the RNA extraction batch previously shown to affect samples’ quality metrics (**Figure S3**) and explain high percentages of expression variation in several genes (**Figure S8**);
iii) variables highly correlated with the total number of fentanyl sessions and this variable itself, as it didn’t impact on sample QC metrics (**Figure S4**) and had minor contributions on gene expression differences (**Figure S8**), and

bioRxiv preprint doi: https://doi.org/10.1101/2025.11.25.690517; this version posted December 12, 2025. The copyright holder for this preprint (which was not certified by peer review) is the author/funder, who has granted bioRxiv a license to display the preprint in perpetuity. It is made available under aCC-BY 4.0 International license.

iv) for other pairs of correlated variables, we only kept the one with the highest percentages of gene expression variance explained obtained with `getVarianceExplained()` (**Figure S8**).

Then, the fraction of variation in the expression of each gene attributable to each included variable was assessed with a variance partition analysis using `fitExtractVarPartModel()` from *variancePartition* [124], jointly accounting for the contributions of the rest of selected variables to confirm their impacts on gene expression and suitability for DGE (**Figure S8**). Sample variables are defined in **Table S3**.

Differential Gene Expression (DGE) analysis

We assessed DGE for substance and behavior under the empirical Bayes framework of *limma-voom* v3.58.1 [65] pipeline, fitting a linear model to the expression of each gene including as covariates the sample variables that were not correlated between them and that explained high percentages of global gene expression variance (**Supplementary Methods: Exploratory Data Analysis**). The gene-wise *p*-values of the resulting moderated *t*-statistics were adjusted for multiple testing using the Benjamini and Hochberg's (BH) procedure that controls the false discovery rate (FDR) [125]. Genes with FDR adjusted *p*-values below 0.05 were considered as DEGs.

The following were the specific DGE analyses performed and the covariates included to model gene expression in each.

<u>DGE for substance (fentanyl vs. saline) in Hb and Amyg samples</u>

Differential expression between fentanyl vs. saline administration ($Substance$) was tested for each gene i by modeling its gene expression (y_{ij}) across the j samples in Hb as:

$$y_{ij} = \beta_{0i} + \beta_{1i}Substance_j + \beta_{2i}Batch\_RNA\_extraction_j + \beta_{3i}concordMapRate_j + \beta_{4i}RIN_j + \varepsilon_{ij}$$

And in Amyg:

$$y_{ij} = \beta_{0i} + \beta_{1i}Substance_j + \beta_{2i}Batch\_RNA\_extraction_j + \beta_{3i}Batch\_lib\_prep_j + \beta_{4i}overallMapRate_j + \beta_{5i}RIN_j + \varepsilon_{ij}$$

Where the β_i's correspond to the estimated model coefficients of the included covariates for the i-th gene and ε_{ij} the observational-level error term.

Similarly, DGE for behavioral covariates of the rats that self-administered fentanyl was assessed under the following models in each brain region. These behavioral covariates were: the slope of fentanyl intake in each session first hour ($First\_hour\_infusion\_slope$), total fentanyl intake ($Total\_Intake$), and last session fentanyl intake ($Last\_Session\_Intake$). Covariates are defined in **Table S3**.

<u>DGE for 1st hour infusion slope in Hb and Amyg fentanyl samples</u>

- In Hb:
  $$y_{ij} = \beta_{0i} + \beta_{1i}First\_hour\_infusion\_slope_j + \beta_{2i}RIN_j + \beta_{3i}RNA\_concentration_j + \beta_{4i}mitoRate_j + \varepsilon_{ij}$$
- In Amyg:
  $$y_{ij} = \beta_{0i} + \beta_{1i}First\_hour\_infusion\_slope_j + \beta_{2i}RIN_j + \beta_{3i}mitoRate_j + \varepsilon_{ij}$$

<u>DGE for total drug intake in Hb and Amyg fentanyl samples</u>

- In Hb:
  $$y_{ij} = \beta_{0i} + \beta_{1i}Total\_Intake_j + \beta_{2i}RIN_j + \beta_{3i}RNA\_concentration_j + \beta_{4i}overallMapRate_j + \varepsilon_{ij}$$

bioRxiv preprint doi: https://doi.org/10.1101/2025.11.25.690517; this version posted December 12, 2025. The copyright holder for this preprint (which was not certified by peer review) is the author/funder, who has granted bioRxiv a license to display the preprint in perpetuity. It is made available under aCC-BY 4.0 International license.

- In Amyg:
  $y_{ij} = \beta_{0i} + \beta_{1i}Total\_Intake_j + \beta_{2i}RIN_j + \beta_{3i}mitoRate_j + \varepsilon_{ij}$

DGE for last session intake in Hb and Amyg fentanyl samples

- In Hb:
  $y_{ij} = \beta_{0i} + \beta_{1i}Last\_Session\_Intake_j + \beta_{2i}RIN_j + \beta_{3i}RNA\_concentration_j + \beta_{4i}mitoRate_j + \varepsilon_{ij}$
- In Amyg:
  $y_{ij} = \beta_{0i} + \beta_{1i}Last\_Session\_Intake_j + \beta_{2i}RIN_j + \beta_{3i}totalAssignedGene_j + \beta_{4i}concordMapRate_j + \varepsilon_{ij}$

Functional enrichment analysis

Gene sets annotated in Gene Ontology (GO) [126] terms and Kyoto Encyclopedia of Genes and Genomes (KEGG) [127] pathways that were significantly overrepresented among our Hb and Amyg DEGs for substance were found with hypergeometric tests implemented in *clusterProfiler* v4.10.0 [66] using `compareCluster()`. The complete set of expressed genes that were assessed for DGE and with available Entrez gene IDs (n=14,066 genes) was taken as the background gene set. The obtained gene set *p*-values were FDR-adjusted [125].

Cell type enrichment analysis

Marker genes for 1) main cell types and and inhibitory neuronal subtypes in control rat Amyg [54], 2) cell types at fine and broad resolutions in the human Hb-enriched epithalamus [52] and human Amyg [53] of neurotypical control donors donors, and 3) all and the Hb neuronal cell subpopulations in the Hb complex of control mice [51] were obtained using normalized and filtered sn/scRNA-seq data. Markers were found implementing the *MeanRatio* method of *DeconvoBuddies* v0.99.0 [67]. Cell types with less than 10 cells were discarded from marker finding analysis.

Briefly, *MeanRatio* defines as cell type markers those genes with the greatest mean expression in the target cell type compared to any other cell type, computing for each gene the ratio between the mean expression in the target cell type, and the highest mean expression among the non-target cell types (i.e. the *MeanRatio*) [67]. The top 100 or 50 (human Hb [52]) marker genes with *MeanRatios* >1 were used (**Table S11**, **Table S12**, **Table S13**, **Table S14**).

Then, rat orthologs of human and mouse cell type marker genes were obtained using *biomaRt* v2.56.1 [68] under the GRCh38 and GRCm39 genome versions for human and mouse, respectively. The sets of cell type-specific markers in rat were assessed for their enrichment among all, up-, and down-regulated fentanyl vs. saline DEGs in rat Hb and Amyg based on the one-sided Fisher's exact test. Expressed genes assessed for DGE were considered the background gene set (n=16,708 genes).

Generalized Gene-Set Analysis of GWAS data

MAGMA v1.10 [69] was run to assess the joint association of genes in each set of all, up-, and down-regulated Hb and Amyg DEGs with multiple SUDs and psychiatric disorders. Briefly, *MAGMA* was provided as input the summary statistics of genome-wide human Single Nucleotide Polymorphisms (SNPs) from six GWASes: schizophrenia (SCZ) [60], Panic Disorder (PD) [59], Opioid Use Disorder (OUD) [56], Substance Use Disorder (SUD) [55], and Major Depressive Disorder (MDD) [57,58] .

bioRxiv preprint doi: https://doi.org/10.1101/2025.11.25.690517; this version posted December 12, 2025. The copyright holder for this preprint (which was not certified by peer review) is the author/funder, who has granted bioRxiv a license to display the preprint in perpetuity. It is made available under aCC-BY 4.0 International license.

For each GWAS, autosomal SNPs were first mapped onto human genes based on the same human genome reference build used in each study (either GRCh37 or GRCh38; hg19 or hg38). For the gene-level analysis SNP *p*-values were used to compute gene-level *p*-values for their association with the phenotype through the SNP-wise mean Z-statistics method. Given that most of the ancestry composition of all examined GWASes was of European Ancestry, the 1000 Genomes European Phase 3 panel [70] was used as the reference dataset to account for linkage disequilibrium between SNPs.

For gene-set analysis, the human orthologs of rat Hb and Amyg DEGs from each set (all, up-, and down-regulated) were first retrieved from Ensembl release 112 (mRatBN7.2) [128] using *biomaRt* v2.61.1 [68]. Then a competitive positive one-sided gene-set analysis was implemented on such sets of human orthologs to assess their association with the GWAS phenotype based on gene-level associations.

bioRxiv preprint doi: https://doi.org/10.1101/2025.11.25.690517; this version posted December 12, 2025. The copyright holder for this preprint (which was not certified by peer review) is the author/funder, who has granted bioRxiv a license to display the preprint in perpetuity. It is made available under aCC-BY 4.0 International license.

Supplementary Figures

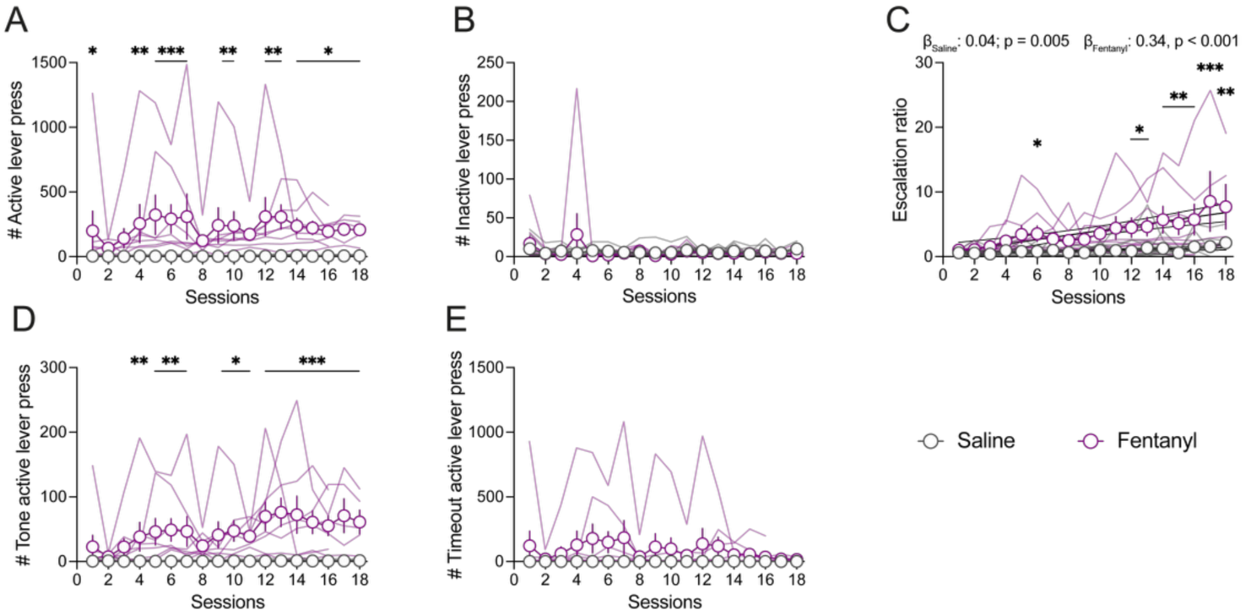

Figure S1: Additional behavior metrics from LgA sessions. Mean number of total (**A**) active (two-way RM ANOVA, substance effect: $F_{1,17}$ = 11.55, p = 0.003; session effect: $F_{17,273}$ = 1.44, p = 0.115; substance x session interaction: $F_{17,273}$ = 1.4, p = 0.133; Student Newman-Keuls post hoc pairwise comparison; ANCOVA substance slope difference: $F_{1,322}$ = 32.07, p < 0.001) and (**B**) inactive lever presses per LgA session for saline and fentanyl rats (two-way RM ANOVA, substance effect: $F_{1,17}$ < 1, p = 0.913; session effect: $F_{17,273}$ = 1.27, p = 0.214; substance x session interaction: $F_{17,273}$ = 1.2, p = 0.265; ANCOVA substance slope difference: $F_{1,322}$ = 32.07, p < 0.001). (**C**) Escalation ratio, an alternative metric to quantify infusion escalation across sessions. This ratio is calculated by normalizing each rat's LgA infusion counts relative to their infusion count on the first LgA session (two-way RM ANOVA, substance effect: $F_{1,17}$ = 8.49, p = 0.01; session effect: $F_{17,273}$ = 4.66, p < 0.001; substance x session interaction: $F_{17,273}$ = 2.8, p < 0.001; ANCOVA substance slope difference: $F_{1,322}$ = 22.88, p < 0.001). (**D**) Number of active lever presses performed during the 2.8 second infusion and tone presentation period (two-way RM ANOVA, substance effect: $F_{1,17}$ = 19.68, p < 0.001; session effect: $F_{17,273}$ = 2.9, p < 0.001; substance x session interaction: $F_{17,273}$ = 2.8, p < 0.001). (**E**) Number of active lever presses performed during the 20 second timeout period (two-way RM ANOVA, substance effect: $F_{1,17}$ = 3.25, p = 0.089; session effect: $F_{17,273}$ = 1.4, p = 0.132; substance x session interaction: $F_{17,273}$ = 1.4, p = 0.13). Data shown as mean across rats ± SEM, superimposed with individual rat data points. Black lines represent linear regression with 95% confidence intervals. Saline: n = 11 rats; Fentanyl: n = 8 rats. *p < 0.05; **p < 0.01; ***p < 0.001.

bioRxiv preprint doi: https://doi.org/10.1101/2025.11.25.690517; this version posted December 12, 2025. The copyright holder for this preprint (which was not certified by peer review) is the author/funder, who has granted bioRxiv a license to display the preprint in perpetuity. It is made available under aCC-BY 4.0 International license.

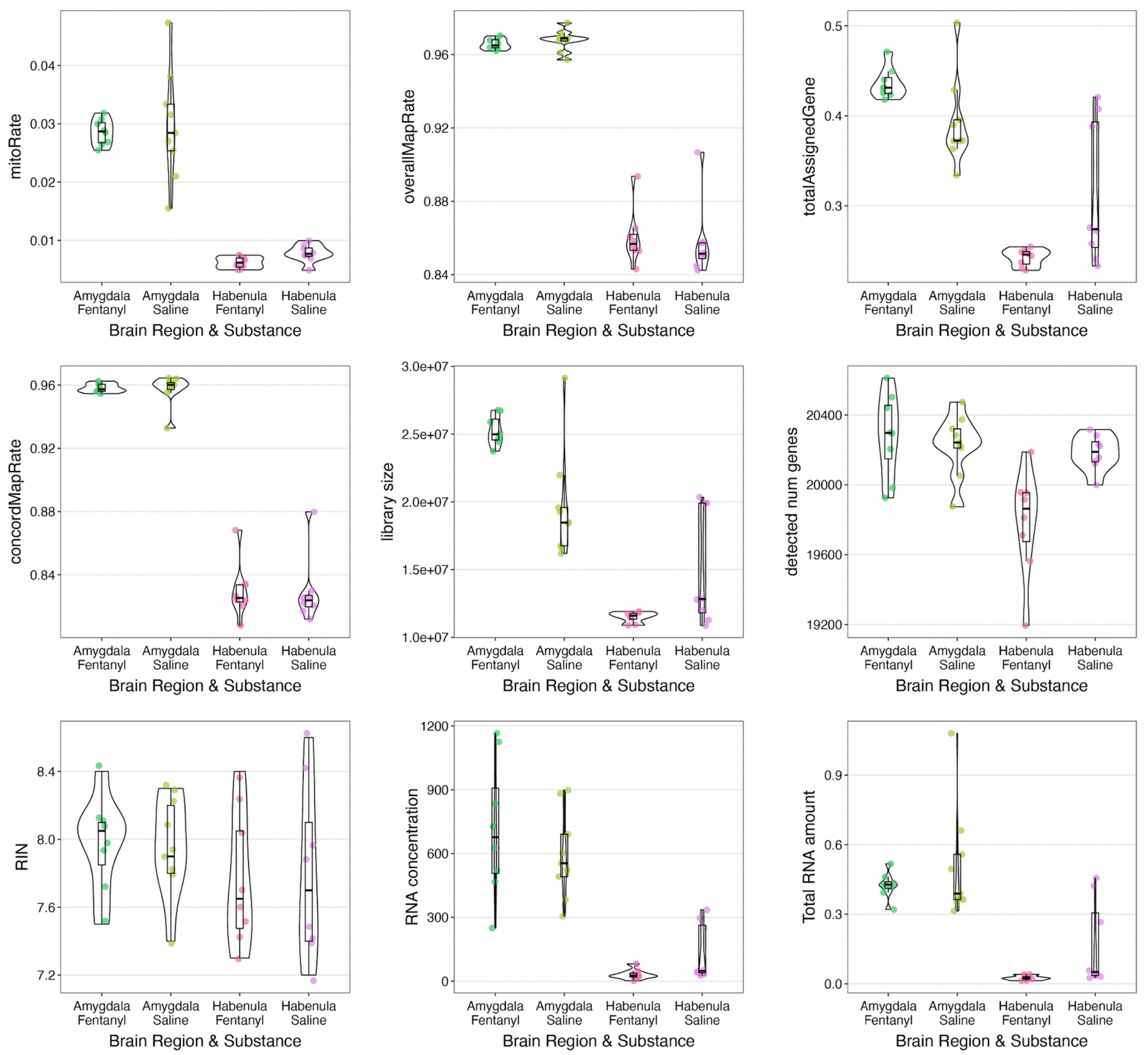

Figure S2: Quality control metrics for Hb and Amyg samples. Comparison of the QC metrics examined in this study for habenula and amygdala fentanyl and saline samples. Note that different Illumina library preparation kits were used for each brain region, thus confounding brain region and kit differences, which motivated independent analyses for each brain region. See **Table S3** for the description of these QC metrics.

bioRxiv preprint doi: https://doi.org/10.1101/2025.11.25.690517; this version posted December 12, 2025. The copyright holder for this preprint (which was not certified by peer review) is the author/funder, who has granted bioRxiv a license to display the preprint in perpetuity. It is made available under aCC-BY 4.0 International license.

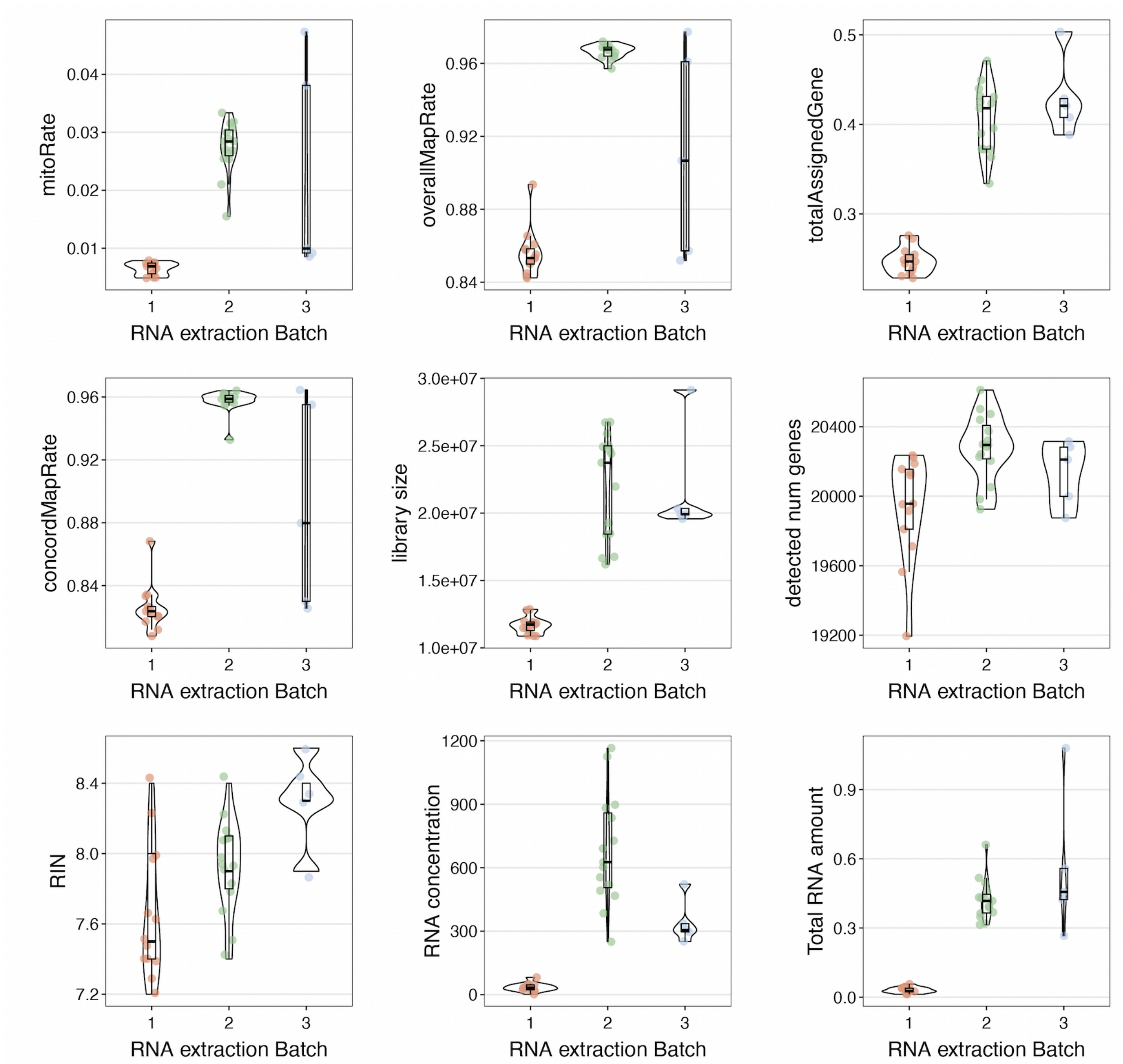

Figure S3: Quality control metrics for samples across RNA extraction batches. Comparison of QC metrics of samples from the first (only Hb samples), second (only Amyg samples), and third batch for RNA extraction (additional Hb and Amyg samples). See **Table S3** for the description of these QC metrics.

bioRxiv preprint doi: https://doi.org/10.1101/2025.11.25.690517; this version posted December 12, 2025. The copyright holder for this preprint (which was not certified by peer review) is the author/funder, who has granted bioRxiv a license to display the preprint in perpetuity. It is made available under aCC-BY 4.0 International license.

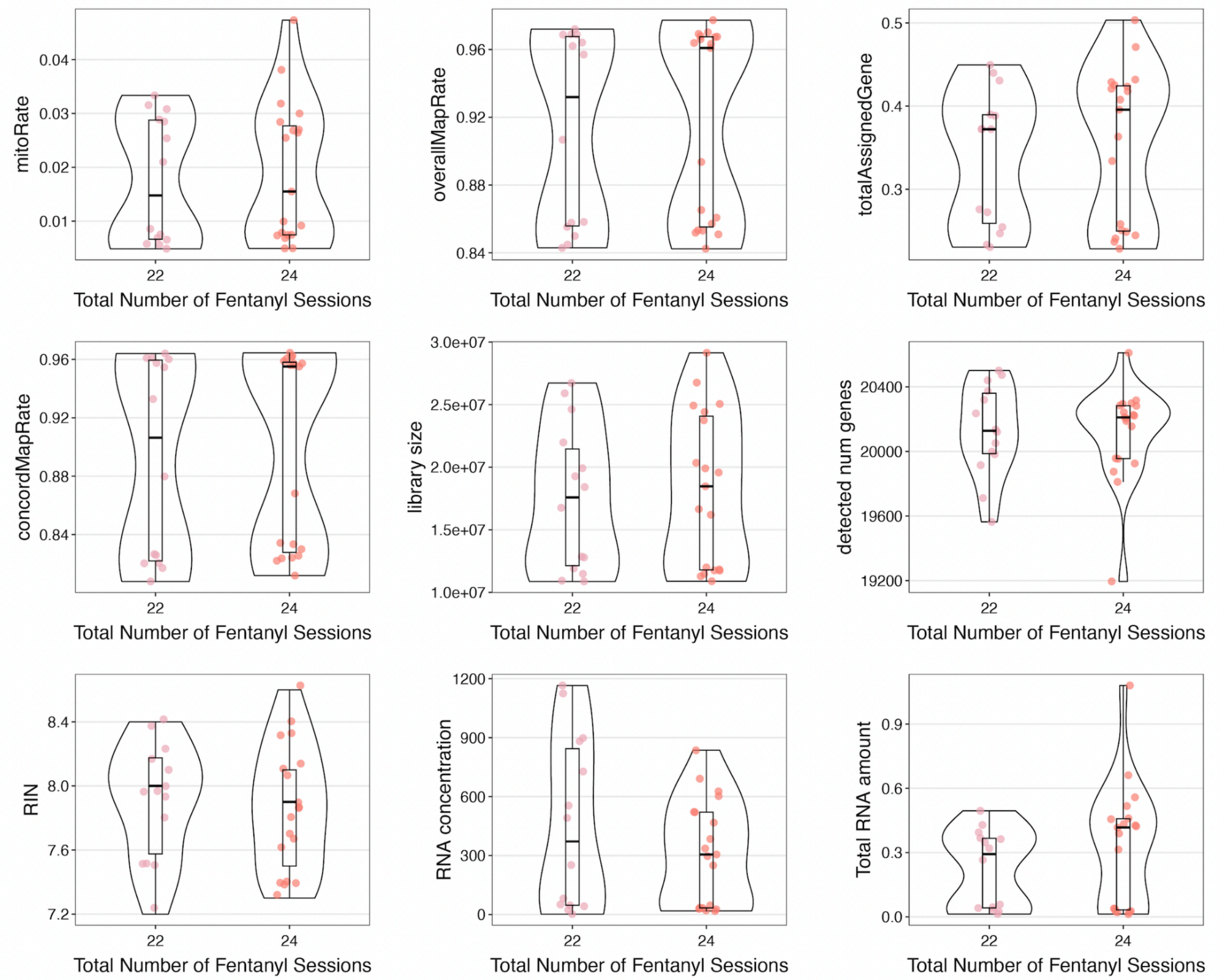

Figure S4: Quality control metrics for samples across total number of self-administration sessions. Comparison of QC metrics for (Hb and Amyg) samples from rats who had 22 and 24 total (fentanyl or saline) self-administration sessions. See **Table S3** for the description of these QC metrics.

bioRxiv preprint doi: https://doi.org/10.1101/2025.11.25.690517; this version posted December 12, 2025. The copyright holder for this preprint (which was not certified by peer review) is the author/funder, who has granted bioRxiv a license to display the preprint in perpetuity. It is made available under aCC-BY 4.0 International license.

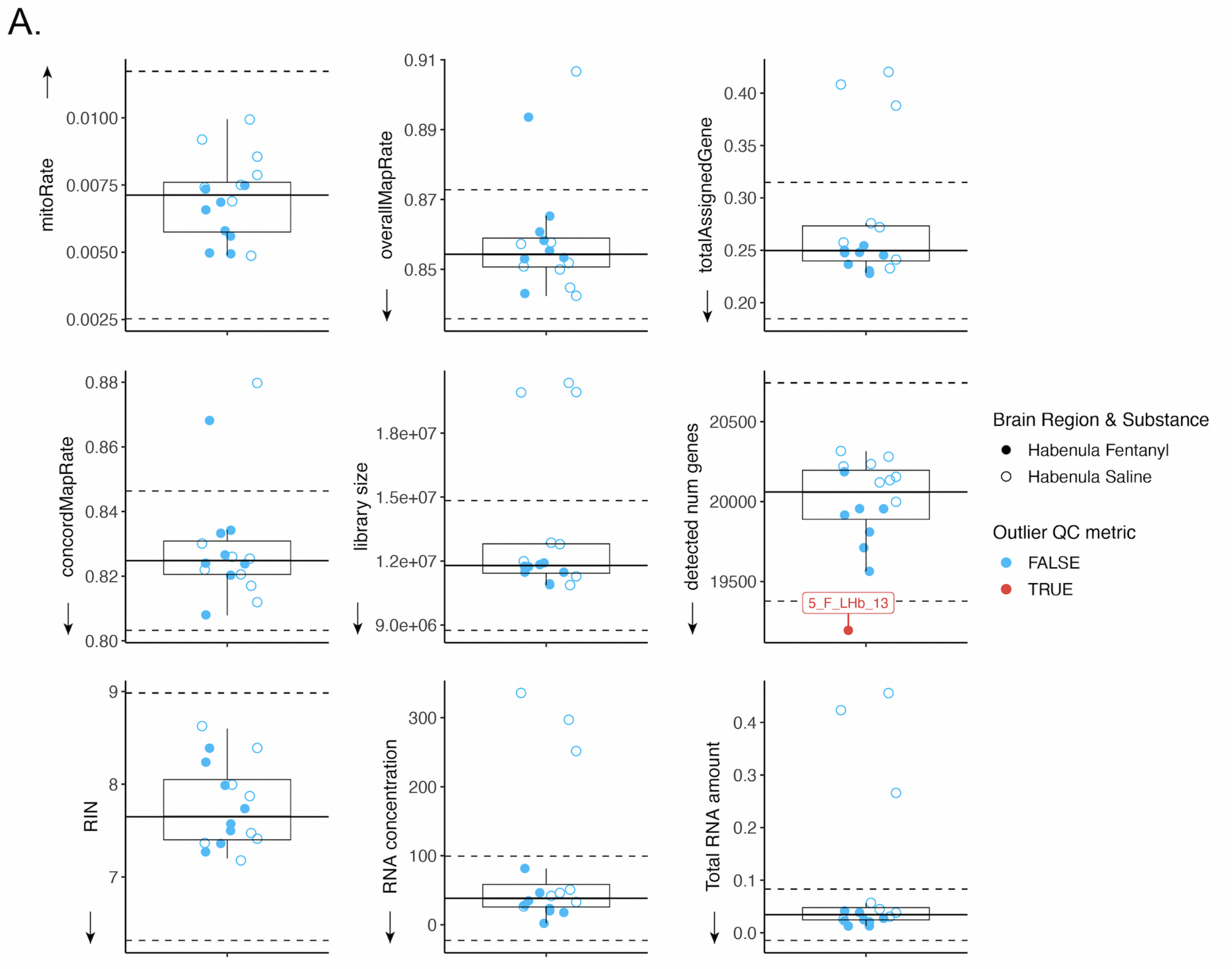

bioRxiv preprint doi: https://doi.org/10.1101/2025.11.25.690517; this version posted December 12, 2025. The copyright holder for this preprint (which was not certified by peer review) is the author/funder, who has granted bioRxiv a license to display the preprint in perpetuity. It is made available under aCC-BY 4.0 International license.

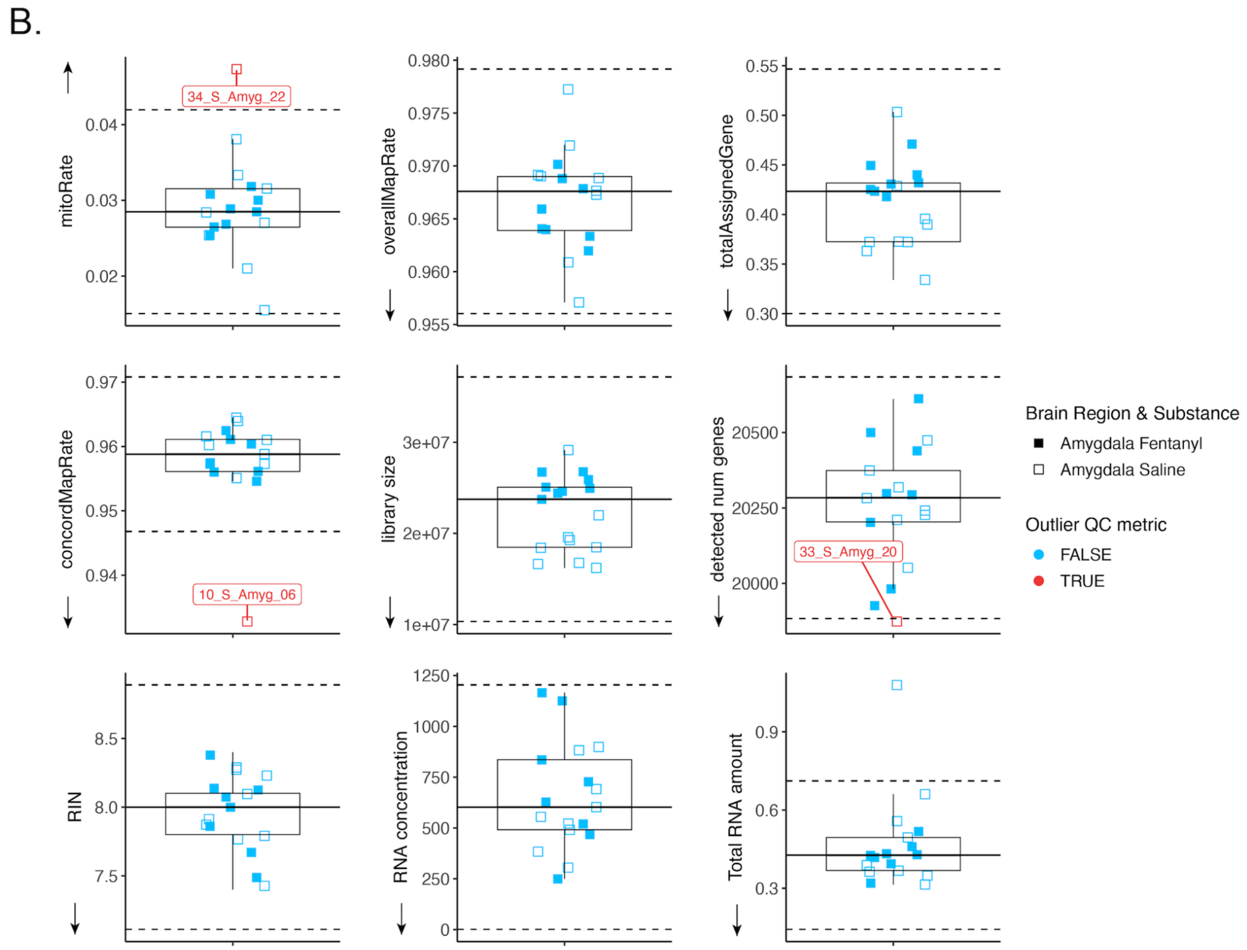

Figure S5: Low-quality sample identification. Detection of low-quality metrics for (**A**) Hb and (**B**) Amyg fentanyl (filled circles/squares) and saline (empty circles/squares) samples. QC metric outliers (in red) were identified as those being 3 median-absolute-deviations (MAD; dotted lines) away from the median (solid line). Only lower outliers were considered poor-quality for all QC metrics except `mitoRate`, for which higher outliers were considered instead (indicated by arrows). Samples with outlier QC metrics are labeled and were subjected to further evaluation in downstream analyses (**Figure S7**). See **Table S3** for the description of these QC metrics.

bioRxiv preprint doi: https://doi.org/10.1101/2025.11.25.690517; this version posted December 12, 2025. The copyright holder for this preprint (which was not certified by peer review) is the author/funder, who has granted bioRxiv a license to display the preprint in perpetuity. It is made available under aCC-BY 4.0 International license.

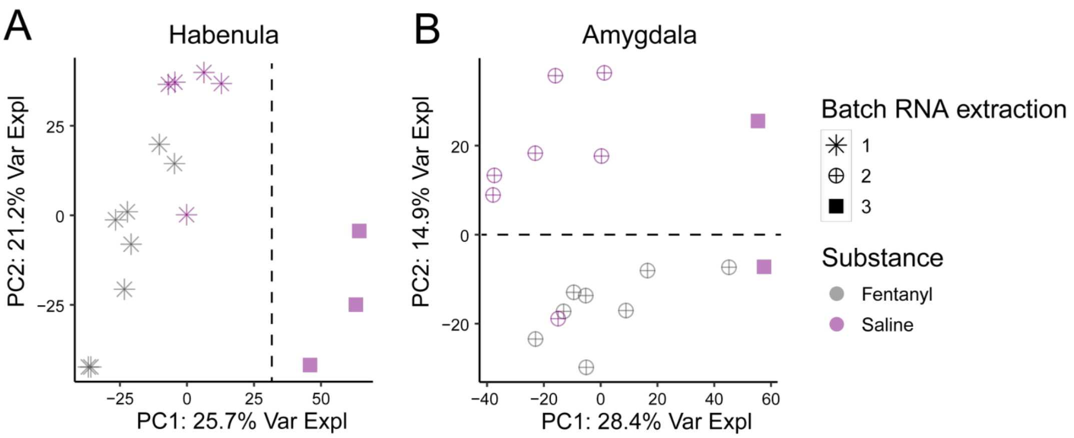

Figure S6: Principal Component Analysis. PC1 vs. PC2 for gene expression in (**A**) Hb and (**B**) Amyg samples. Percentages of variance explained by each PC are indicated on the axes. Samples are shaped by RNA extraction batch and colored by substance.

bioRxiv preprint doi: https://doi.org/10.1101/2025.11.25.690517; this version posted December 12, 2025. The copyright holder for this preprint (which was not certified by peer review) is the author/funder, who has granted bioRxiv a license to display the preprint in perpetuity. It is made available under aCC-BY 4.0 International license.

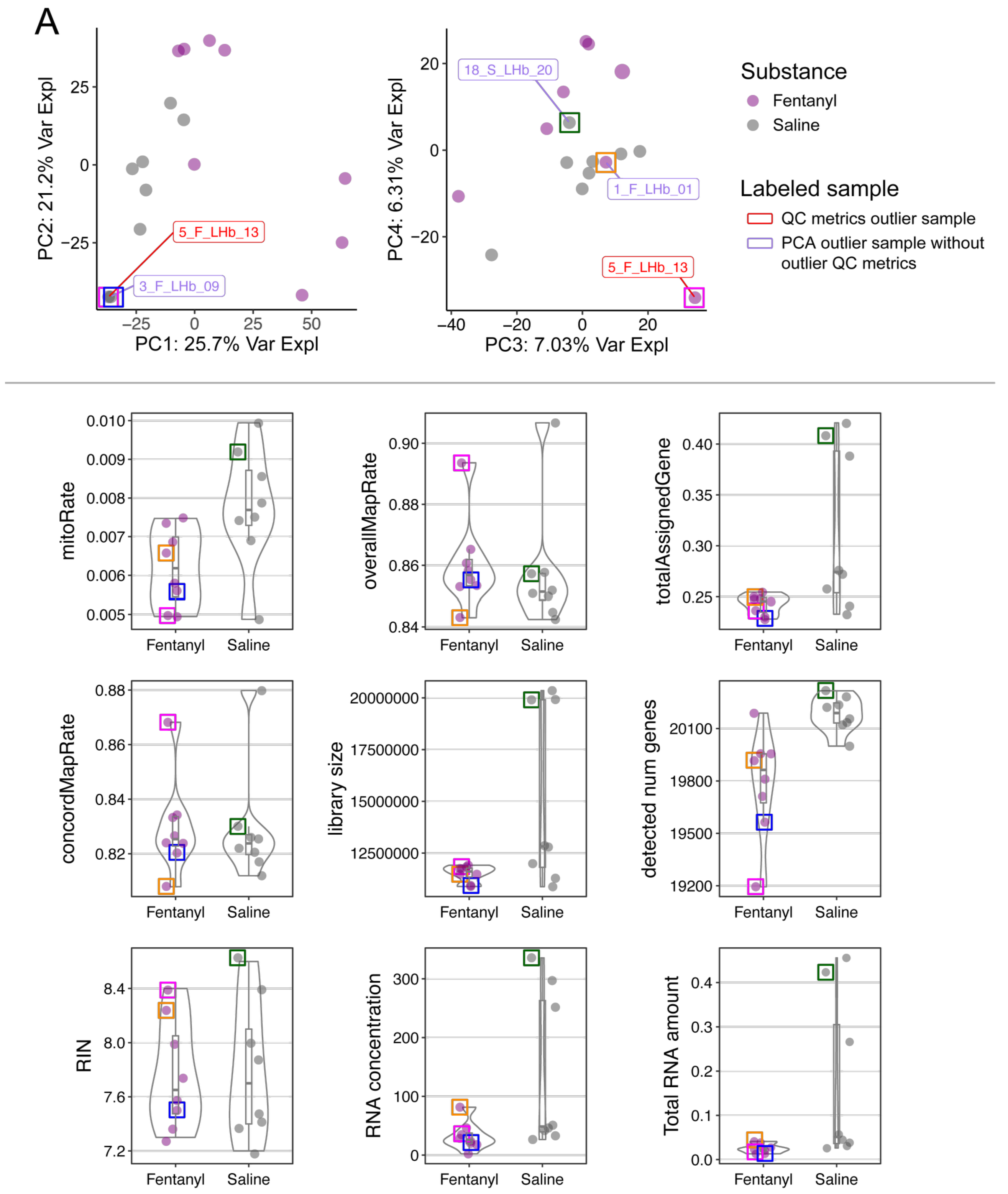

bioRxiv preprint doi: https://doi.org/10.1101/2025.11.25.690517; this version posted December 12, 2025. The copyright holder for this preprint (which was not certified by peer review) is the author/funder, who has granted bioRxiv a license to display the preprint in perpetuity. It is made available under aCC-BY 4.0 International license.

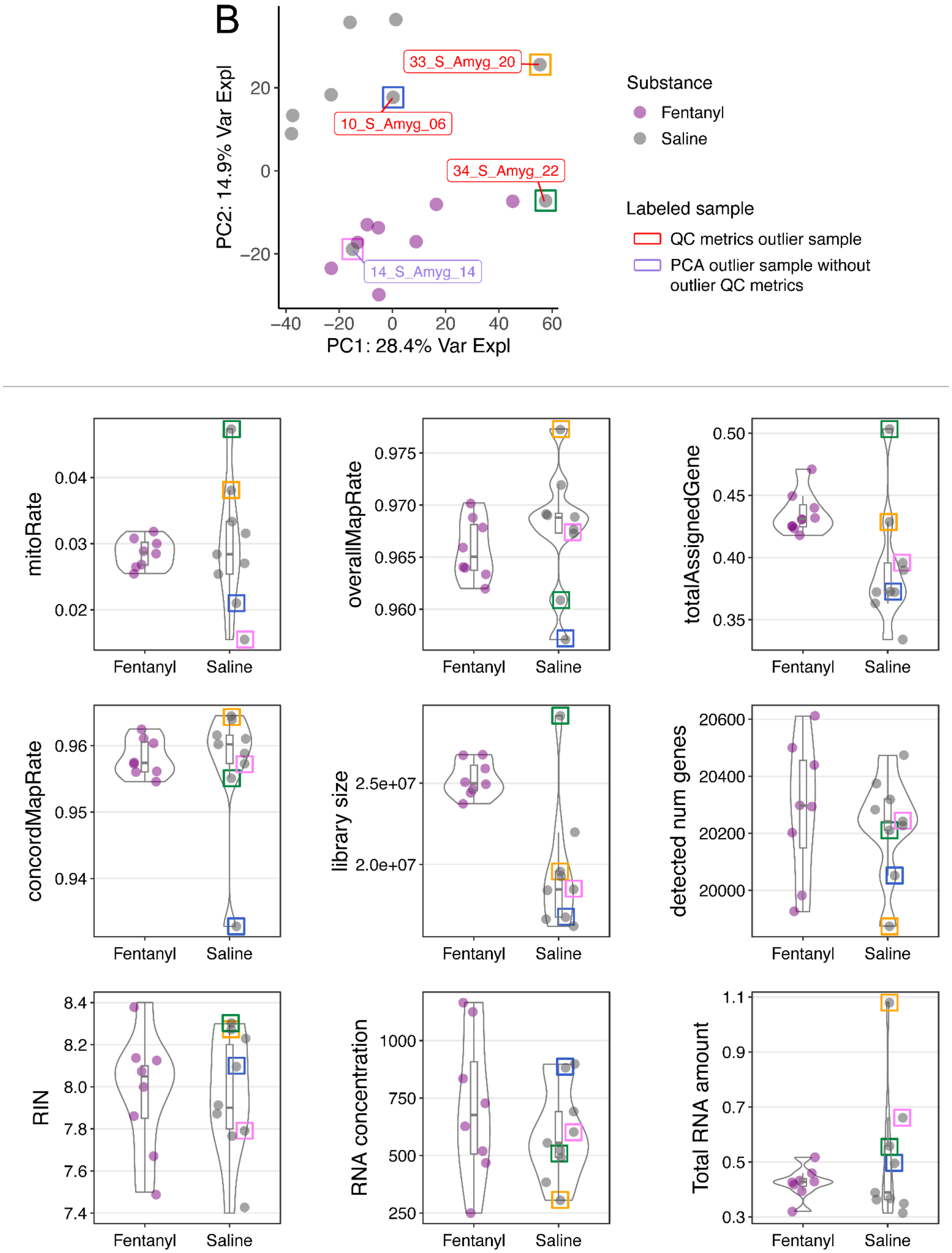

Figure S7: Manual sample quality examination based on PCA. PCx vs. PCy (top) for (**A**) Hb and (**B**) Amyg samples. QC metrics outlier samples are labeled in red (see **Figure S5**); samples segregated from the rest in each PC plot, as well as fentanyl and saline samples closer to samples from the other substance group were considered PCA outlier samples and are labeled in purple. The percentage of variance explained by each PC

bioRxiv preprint doi: https://doi.org/10.1101/2025.11.25.690517; this version posted December 12, 2025. The copyright holder for this preprint (which was not certified by peer review) is the author/funder, who has granted bioRxiv a license to display the preprint in perpetuity. It is made available under aCC-BY 4.0 International license.

is shown on axis labels. For both, QC metrics and PCA outlier samples, all their QC metrics were reexamined (bottom box plots); different colored squares indicate the different outlier samples. Samples in all plots are colored by substance. See **Table S3** for the description of these QC metrics.

bioRxiv preprint doi: https://doi.org/10.1101/2025.11.25.690517; this version posted December 12, 2025. The copyright holder for this preprint (which was not certified by peer review) is the author/funder, who has granted bioRxiv a license to display the preprint in perpetuity. It is made available under aCC-BY 4.0 International license.

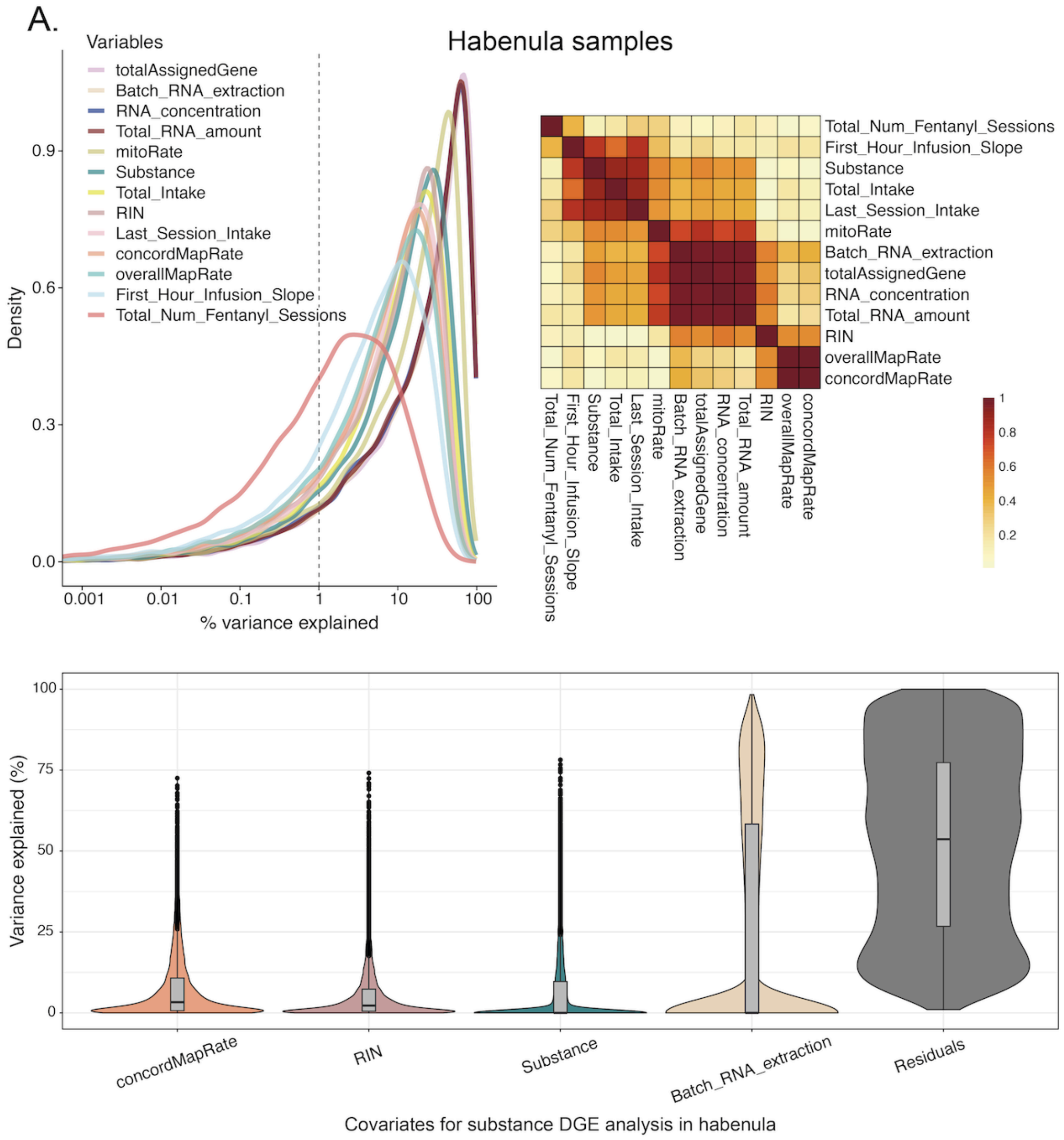

bioRxiv preprint doi: https://doi.org/10.1101/2025.11.25.690517; this version posted December 12, 2025. The copyright holder for this preprint (which was not certified by peer review) is the author/funder, who has granted bioRxiv a license to display the preprint in perpetuity. It is made available under aCC-BY 4.0 International license.

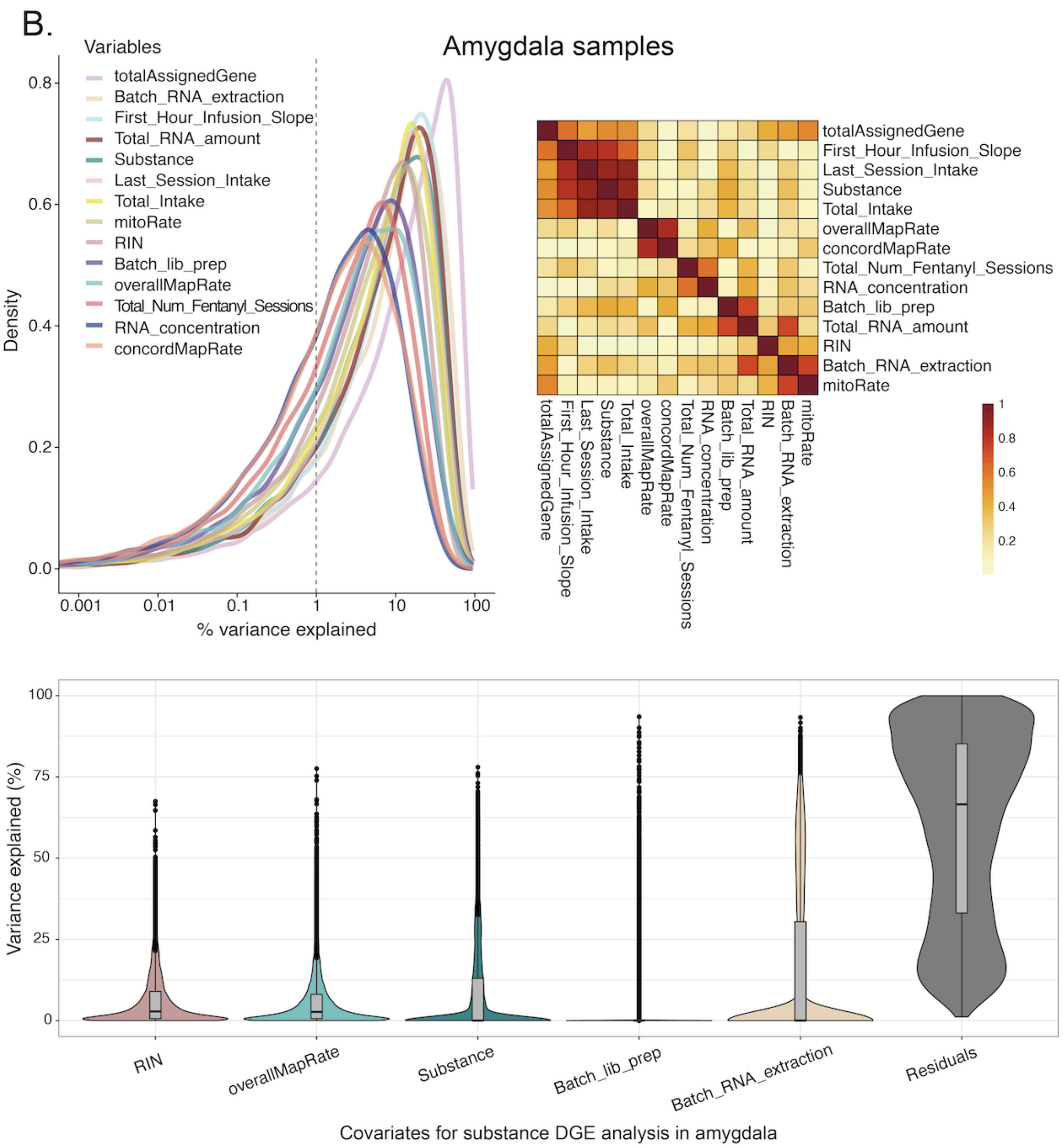

bioRxiv preprint doi: https://doi.org/10.1101/2025.11.25.690517; this version posted December 12, 2025. The copyright holder for this preprint (which was not certified by peer review) is the author/funder, who has granted bioRxiv a license to display the preprint in perpetuity. It is made available under aCC-BY 4.0 International license.

C.

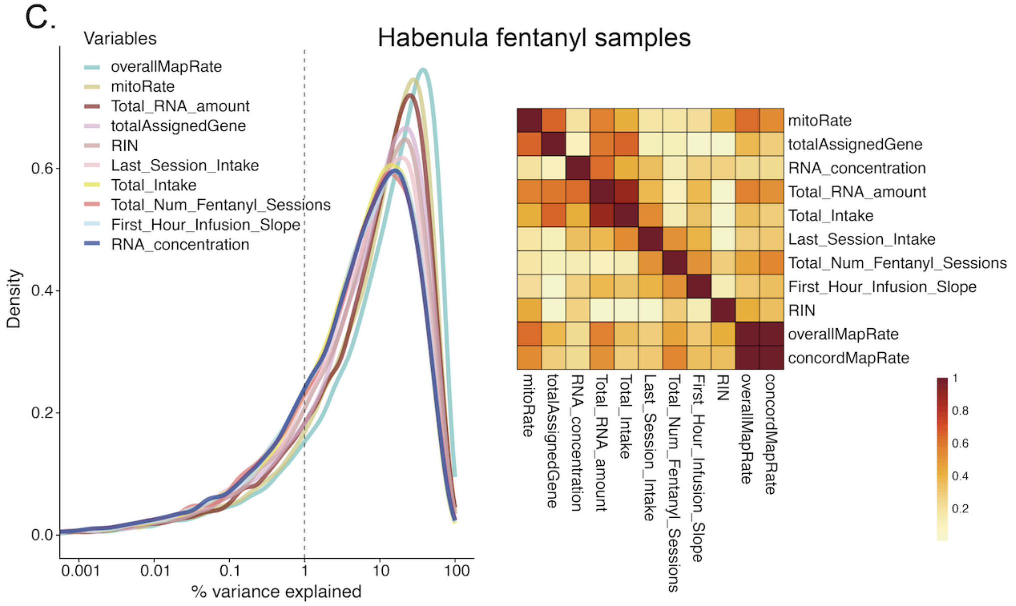

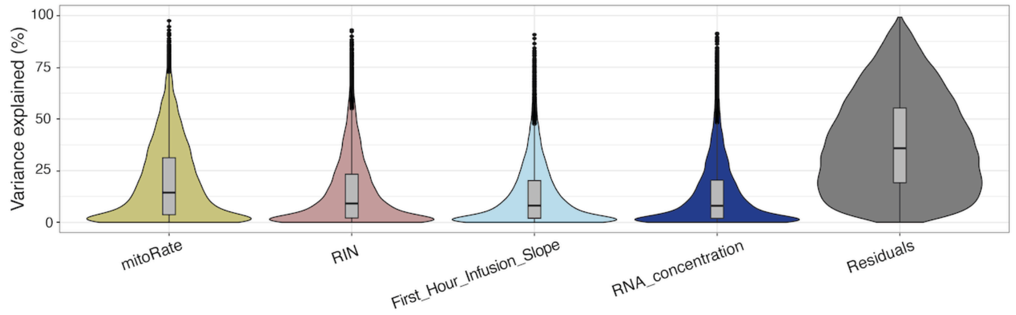

Covariates for first hour infusion slope DGE analysis in habenula

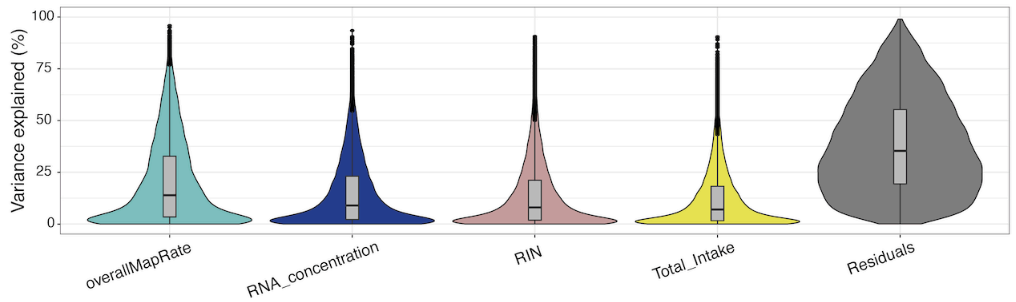

Covariates for total intake DGE analysis in habenula

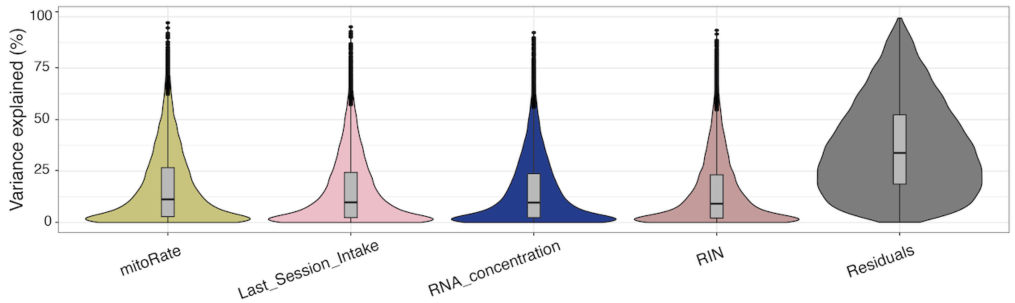

Covariates for last session intake DGE analysis in habenula

bioRxiv preprint doi: https://doi.org/10.1101/2025.11.25.690517; this version posted December 12, 2025. The copyright holder for this preprint (which was not certified by peer review) is the author/funder, who has granted bioRxiv a license to display the preprint in perpetuity. It is made available under aCC-BY 4.0 International license.

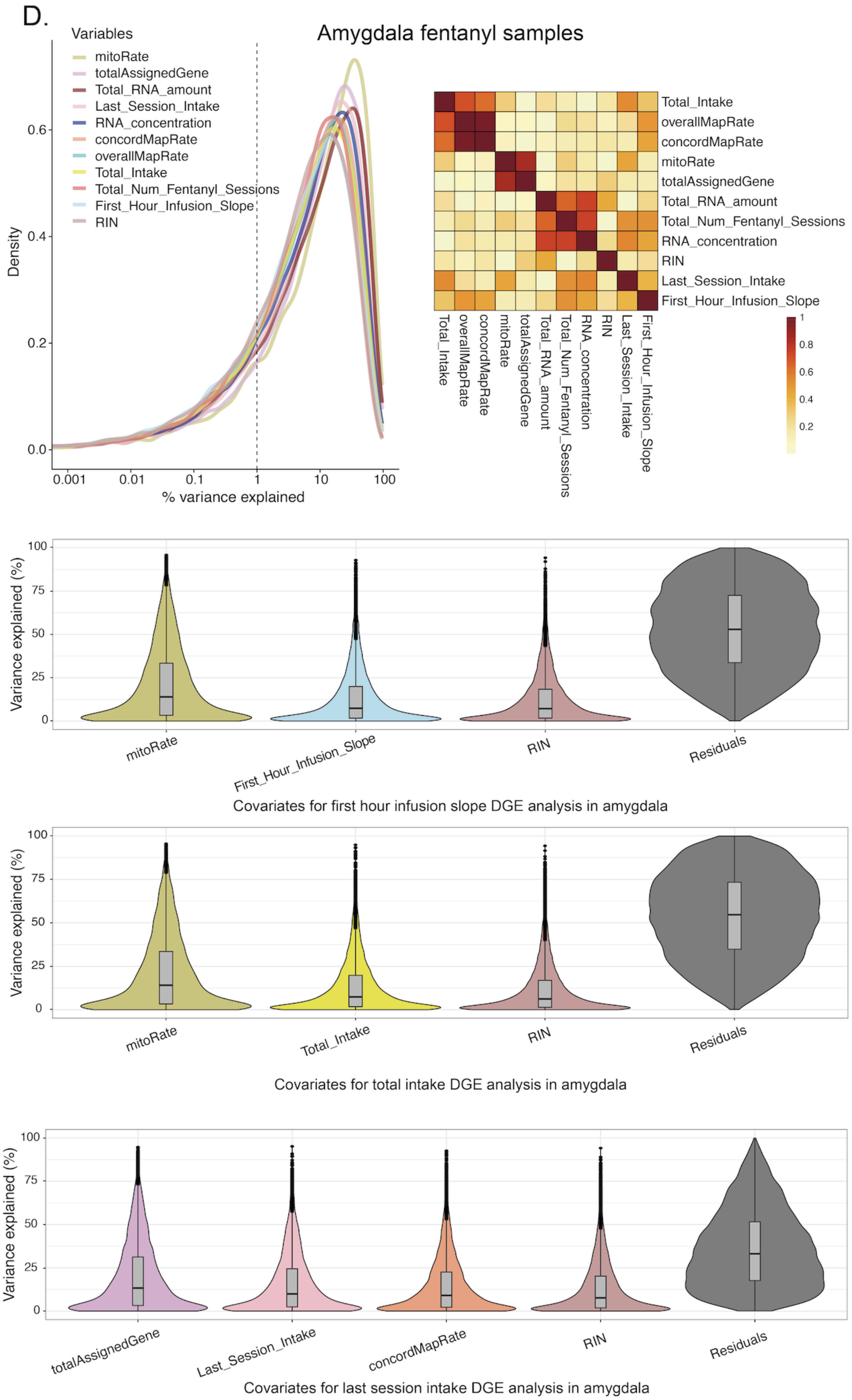

Figure S8: Sample-level covariate selection for DGE analysis. Gene expression variance partition analysis in **A.** Hb (all rats), **B.** Amyg (all rats), **C.** Hb (fentanyl rats only), and **D.** Amyg (fentanyl rats only). Top left:

bioRxiv preprint doi: https://doi.org/10.1101/2025.11.25.690517; this version posted December 12, 2025. The copyright holder for this preprint (which was not certified by peer review) is the author/funder, who has granted bioRxiv a license to display the preprint in perpetuity. It is made available under aCC-BY 4.0 International license.

density plot for the percentages of variance explained in the expression of each gene by each sample-level variable. Top right: canonical correlation between each pair of variables. Variables included in the models for DGE analyses (A-B. for substance, and C-D. for rat behavioral traits) were selected based on their contributions to gene expression variance and correlations with other variables. Bottom: percentage of variance in the expression of each gene explained by each variable included in the DGE model, considering all other included variables in the model (x-axis); variables are ordered by decreasing median percentage of variance explained. Related to **Figure 2**. See **Table S3** for the description of these variables and QC metrics.

bioRxiv preprint doi: https://doi.org/10.1101/2025.11.25.690517; this version posted December 12, 2025. The copyright holder for this preprint (which was not certified by peer review) is the author/funder, who has granted bioRxiv a license to display the preprint in perpetuity. It is made available under aCC-BY 4.0 International license.

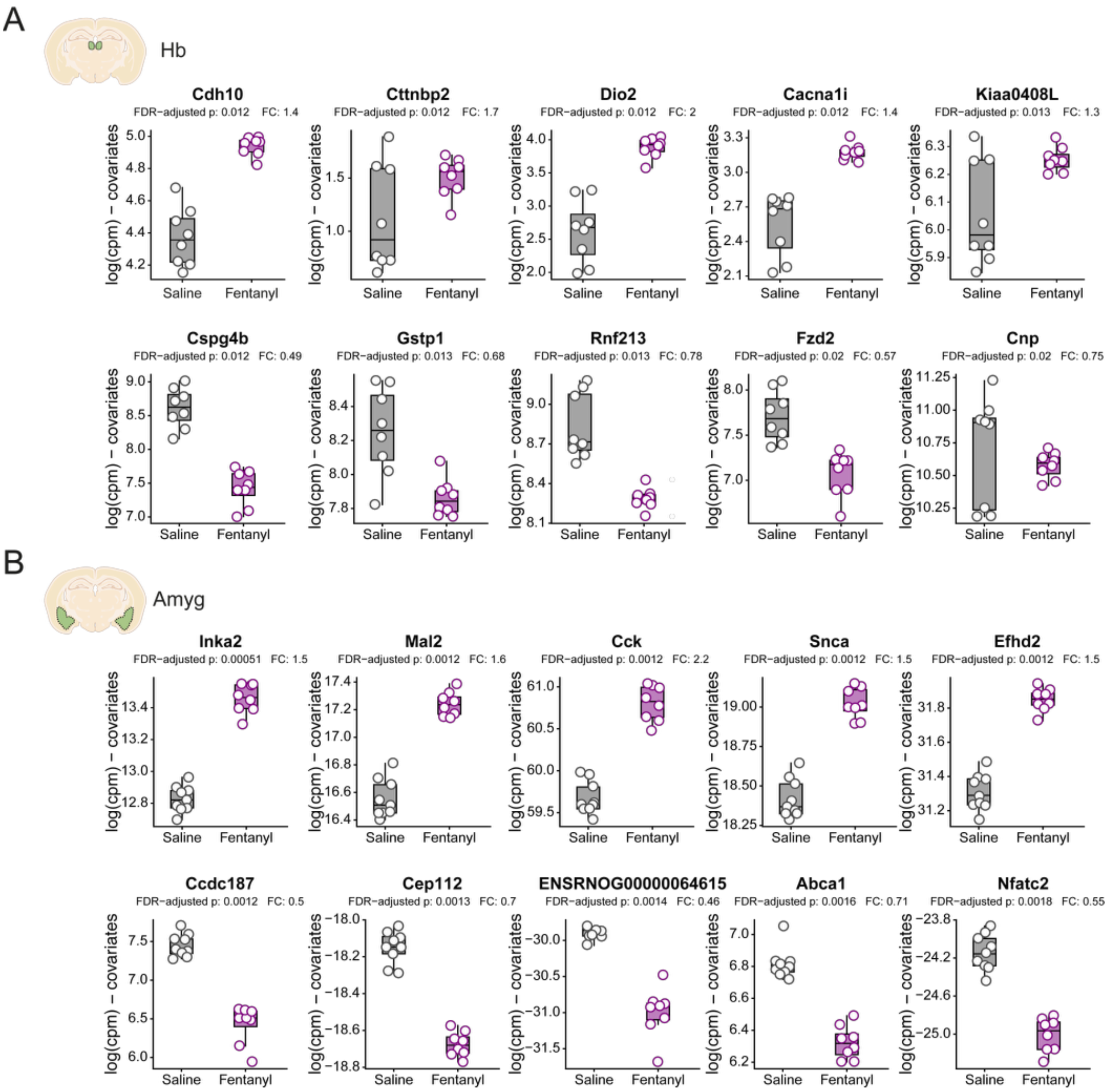

Figure S9: Top 5 differentially expressed genes in Hb and Amyg following chronic LgA fentanyl self-administration. (**A-B**) Box plots showing the expression of the top five up- and down-regulated DEGs for fentanyl vs. saline in Hb (**A**) and Amyg (**B**). Gene expression is given in log_2(CPM) after regressing out covariates. FDR-adjusted p-value and fold change (FC) are shown for each gene. Boxes extend from the 25th to 75th percentiles; lines within the boxes represent the median; whiskers indicate the minimum and maximum values, superimposed with individual rat data points. Fentanyl Hb n = 8; Saline Hb n = 8; Fentanyl Amyg n = 8; Saline Amyg n = 9. Related to **Figure 2**.

bioRxiv preprint doi: https://doi.org/10.1101/2025.11.25.690517; this version posted December 12, 2025. The copyright holder for this preprint (which was not certified by peer review) is the author/funder, who has granted bioRxiv a license to display the preprint in perpetuity. It is made available under aCC-BY 4.0 International license.

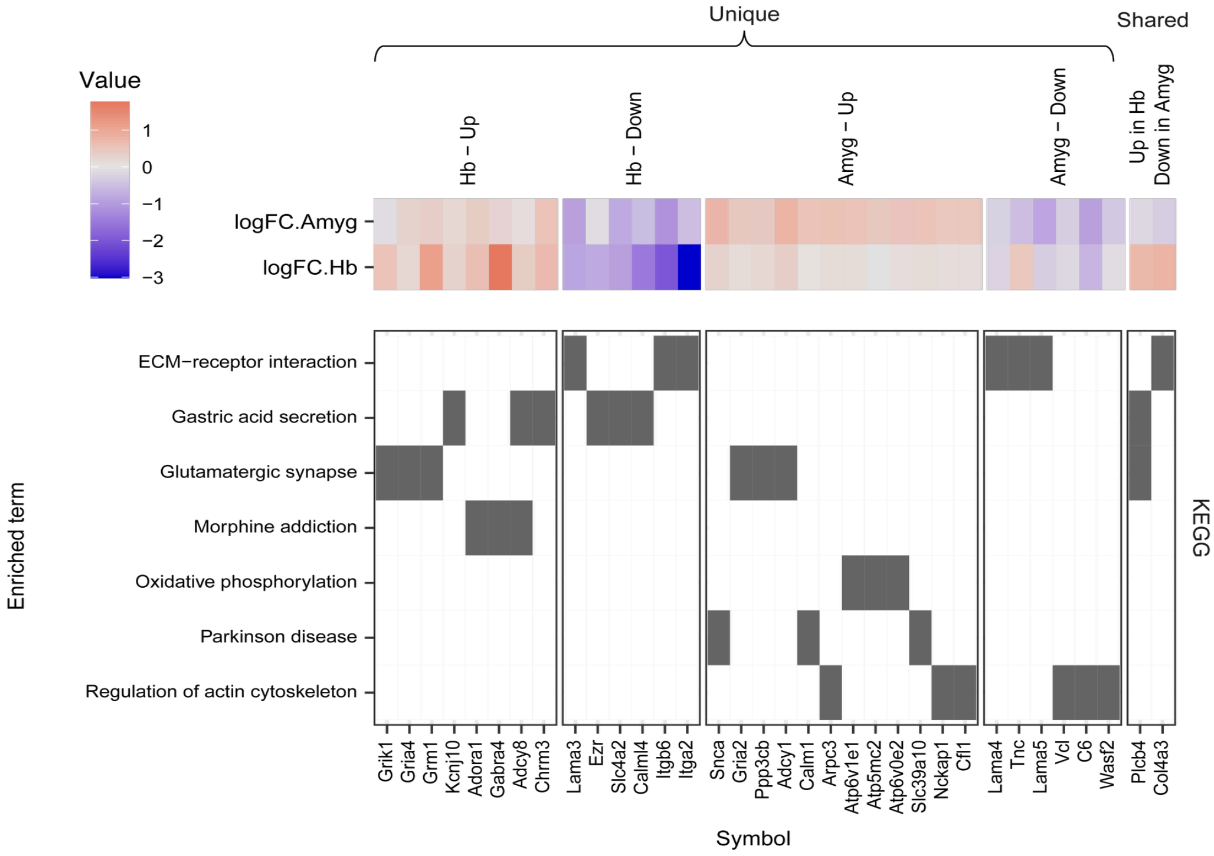

Figure S10: Biological KEGG pathways dysregulated by chronic fentanyl self-administration in Hb and Amyg. Tile plot displays DEG (x-axis) membership to an enriched pathway as a filled tile. Key DEGs from each pathway are shown, categorized by their unique or shared up- and down-regulation in Hb and Amyg. Top heatmap shows DEG mean-centered $\log_2$FC in Hb and Amyg. Related to **Figure 2**, **Table S8**, **Table S9**.

bioRxiv preprint doi: https://doi.org/10.1101/2025.11.25.690517; this version posted December 12, 2025. The copyright holder for this preprint (which was not certified by peer review) is the author/funder, who has granted bioRxiv a license to display the preprint in perpetuity. It is made available under aCC-BY 4.0 International license.

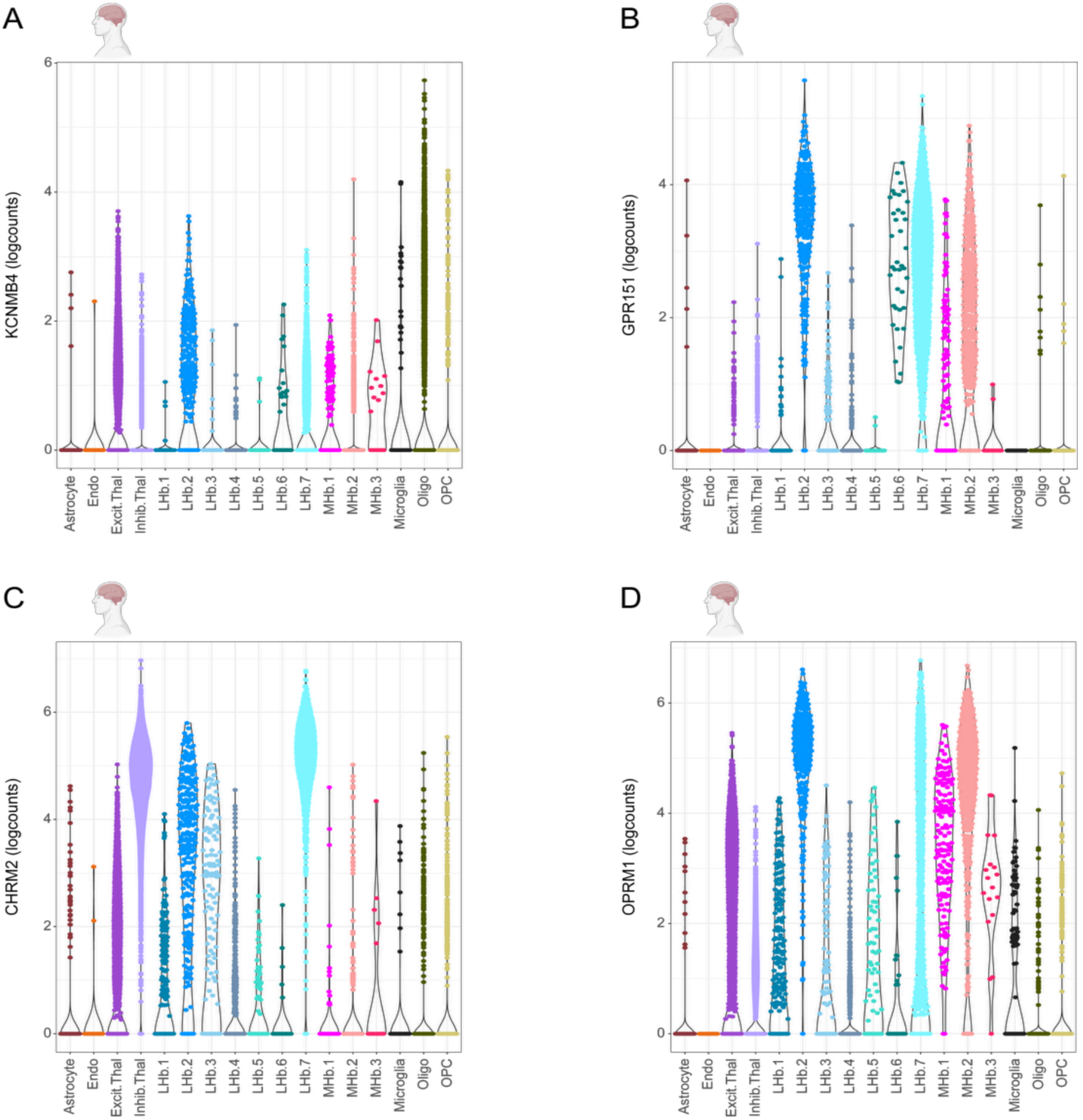

Figure S11: Expression of *KCNMB4*, *GPR151, CHRM2* and *OPRM1* in human habenula cell types. (A-D) Violin plots showing expression of (**A**) *KCNMB4*, (**B**) *GPR151*, (**C**) *CHRM2*, and (**D**) *OPRM1* in human Hb cell types from Yalcinbas et al., 2025 [52]. *Kcnmb4*, *Gpr151*, and *Chrm2* mark the mouse LHb.6 subpopulation identified by Hashikawa et. al. [51], which we found to be enriched in our rat upregulated Hb fentanyl DEGs. These genes are highly expressed in human LHb.2 and LHb.7 subpopulations, which also express *OPRM1*. This suggests that fentanyl-sensitive mouse LHb.6 may be conserved with these *OPRM1*-expressing human LHb.2 and LHb.7 neuronal populations. Related to **Figure 3**.

bioRxiv preprint doi: https://doi.org/10.1101/2025.11.25.690517; this version posted December 12, 2025. The copyright holder for this preprint (which was not certified by peer review) is the author/funder, who has granted bioRxiv a license to display the preprint in perpetuity. It is made available under aCC-BY 4.0 International license.

Supplementary References

118. Di Tommaso P, Chatzou M, Floden EW, Barja PP, Palumbo E, Notredame C. Nextflow enables reproducible computational workflows. Nat Biotechnol. 2017;35:316–319.
119. Kim D, Paggi JM, Park C, Bennett C, Salzberg SL. Graph-based genome alignment and genotyping with HISAT2 and HISAT-genotype. Nat Biotechnol. 2019;37:907–915.
120. Howe K, Dwinell M, Shimoyama M, Corton C, Betteridge E, Dove A, et al. The genome sequence of the Norway rat, Rattus norvegicus Berkenhout 1769. Wellcome Open Res. 2021;6:118.
121. Martin FJ, Amode MR, Aneja A, Austine-Orimoloye O, Azov AG, Barnes I, et al. Ensembl 2023. Nucleic Acids Res. 2023;51:D933–D941.
122. Robinson MD, McCarthy DJ, Smyth GK. edgeR: a Bioconductor package for differential expression analysis of digital gene expression data. Bioinformatics. 2010;26:139–140.
123. McCarthy DJ, Campbell KR, Lun ATL, Wills QF. Scater: pre-processing, quality control, normalization and visualization of single-cell RNA-seq data in R. Bioinformatics. 2017;33:1179–1186.
124. Hoffman GE, Schadt EE. variancePartition: interpreting drivers of variation in complex gene expression studies. BMC Bioinformatics. 2016;17:483.
125. Benjamini Y, Hochberg Y. Controlling the false discovery rate: a practical and powerful approach to multiple testing. Journal of the Royal Statistical Society: Series B (Methodological). 1995;57:289–300.
126. Gene Ontology Consortium. Gene Ontology Consortium: going forward. Nucleic Acids Res. 2015;43:D1049-56.
127. Kanehisa M, Goto S. KEGG: Kyoto encyclopedia of genes and genomes. Nucleic Acids Res. 2000;28:27–30.
128. Dyer SC, Austine-Orimoloye O, Azov AG, Barba M, Barnes I, Barrera-Enriquez VP, et al. Ensembl 2025. Nucleic Acids Res. 2025;53:D948–D957.
